# ZNF703 promotes tumor progression in ovarian cancer by interacting with HE4 and epigenetically regulating PEA15

**DOI:** 10.1186/s13046-020-01770-0

**Published:** 2020-11-27

**Authors:** Shuang Wang, Caixia Wang, Yuexin Hu, Xiao Li, Shan Jin, Ouxuan Liu, Rui Gou, Yuan Zhuang, Qian Guo, Xin Nie, Liancheng Zhu, Juanjuan Liu, Bei Lin

**Affiliations:** 1 Department of Obstetrics and Gynaecology, Shengjing Hospital Affiliated to China Medical University, No. 36, Sanhao Street, Heping District, Liaoning Shenyang, 110004 People’s Republic of China; 2 Key Laboratory of Maternal-Fetal Medicine of Liaoning Province, Key Laboratory of Obstetrics and Gynecology of Higher Education of Liaoning Province, Shenyang, Liaoning China; 3 Department of Obstetrics and Gynecology, West China Second University Hospital, Sichuan University, Chengdu, China; 4 Department of Obstetrics and Gynaecology, Cancer Hospital of China Medical University, Liaoning Cancer Hospital and Institute, Shenyang, Liaoning China

**Keywords:** Ovarian cancer, ZNF703, HE4, PEA15, ChIP-Seq

## Abstract

**Background:**

It is known that the transcription factor zinc finger protein 703 (ZNF703) plays an important role in physiological functions and the occurrence and development of various tumors. However, the role and mechanism of ZNF703 in ovarian cancer are unclear.

**Materials and methods:**

Immunohistochemistry was used to analyze the expression of ZNF703 in ovarian cancer patients and to assess the effect of ZNF703 expression on the survival and prognosis of ovarian cancer patients. ZNF703 overexpression and suppression expression experiments were used to evaluate the effect of ZNF703 on malignant biological behavior of ovarian cancer cells in vitro. Detecting the interaction between HE4 and ZNF703 by immunofluorescence colocalization and coprecipitation, and nuclear translocation. Chromatin immunoprecipitation-sequencing (ChIP-Seq), dual luciferase reporter assay, ChIP-PCR, in vivo model were applied to study the molecular mechanism of ZNF703 affecting the development of ovarian cancer.

**Results:**

ZNF703 was highly expressed in ovarian cancer tissues, and its expression level is related to the prognosis of ovarian cancer patients. In vivo and in vitro experiments confirmed that ZNF703 overexpression/inhibition expression will promoted/inhibited the malignant biological behavior of ovarian cancer. Mechanically, ZNF703 interacted with HE4, and HE4 promoted nuclear translocation of ZNF703. ChIP-Seq identified multiple regulatory targets of ZNF703, of which ZNF703 directly binds to the enhancer region of PEA15 to promote the transcription of PEA15 and thereby promoted the proliferation of cancer cells.

**Conclusion:**

The results showed that ZNF703 as an oncogene played an important role in the epigenetic modification of ovarian cancer proliferation, and suggested that ZNF703 as a transcription factor may become a prognostic factor and a potential therapeutic target for ovarian cancer.

**Supplementary Information:**

The online version contains supplementary material available at 10.1186/s13046-020-01770-0.

## Background

Ovarian cancer is a gynecological malignant tumor that has the highest mortality rate. At present, surgery and chemotherapy are still the main treatment methods for ovarian cancer. However, as 70% of the patients are in the advanced stage at the time of diagnosis, their extensive pelvic and abdominal metastases and chemotherapy resistance often result in poor prognosis and a low survival rate. The five-year survival rate for ovarian cancer patients is 48.6% [1]. Although progress has been made in the diagnosis and treatment of ovarian cancer, further research is needed to clarify the causative factors and develop effective measures for early detection and treatment [2, 3].

Zinc finger proteins are transcription factors (TFs) widely found in eukaryotes; they can regulate cancer cell proliferation, differentiation, apoptosis, invasion, and migration. ZNF703 is a member of the NET/Nlz family of transcription factors with a “finger” domain [4], located in the cytoplasm and nucleus. Studies have shown that ZNF703 regulated embryonic development processes, such as the differentiation of the vertebrate hindbrain into rhomboids, and the formation of the Xenopus neural crest, under physiological conditions [5, 6]. ZNF703 was originally found from the abnormal amplification of the 8p11–12 region of the human chromosome, which led to irregular gene expression and the occurrence and progression of breast cancer [7, 8]. Recent studies confirmed that the expression of ZNF703 promoted tumor progression and was abnormally increased in breast [9], lung [10], and gastric cancers [11].

An anti-apoptotic factor composed of 131 amino acids, PEA15 was originally found in primary cultured astrocytes [12]. The first 80 amino acids form a standardized “death effect zone (DED)”, whereas the latter 51 amino acids form an irregularly structured C-terminal tail (containing phosphorylation sites) [12]. PEA15 is primarily involved in cell proliferation, differentiation, cell signal transduction, apoptosis regulation, the cell cycle, and glucose transport processes [13, 14]. It is closely related to tumor occurrence, development, and metastasis, and its expression is up-regulated in various tumors [15–17].

We initially discovered ZNF703 in the sequencing results we obtained while investigating HE4’s protein-protein interactions using the yeast two-hybrid (Y2H) assay. The aim of the present study was to investigate the epigenetic regulation of ZNF703 in ovarian cancer, reveal the pathways involved, the underlying mechanism, and determine how this affects ovarian cancer progression and the survival time of patients.

## Materials and methods

### Specimen source and clinical data

The specimens included 98 ovarian epithelial malignant tumor samples (ovarian cancer group), 15 ovarian epithelial borderline tumor samples (borderline group), 14 ovarian epithelial benign tumor samples (benign group), and 12 normal ovarian tissue samples (normal group). The average age of all the patients included in this study was 53.24 years (19–84 years). The ovarian tissue samples we analyzed were selected from the archived wax blocks of the surgical specimens of inpatients in our hospital from 2008 to 2014. The patients the samples were derived from had not undergone chemotherapy, radiotherapy or hormone therapy before surgery, and their complete clinical information was available. The clinical surgical pathological staging was carried out according to the standards set by the International Federation of Obstetrics and Gynecology (FIGO) in 2009.

### Immunohistochemistry and immunocytochemistry

Continuous 5 μm-thick paraffin sections were used for immunohistochemistry. The concentration of the monoclonal antibody used against ZNF703 was 1:50 (Santa Cruz, sc-271,896). After dewaxing, the samples were permeated using immunostaining permeation solution (Triton X-100, P0096, Beyotime), and the staining method was carried out using the kit for the streptavidin-peroxidase connection (SP) method according to the instructions of the manufacturer. A positive result was indicated by the presence of brown particles in the nucleus and cytoplasm. According to the coloring intensity, the samples were divided into noncolored, light yellow, brown yellow and brown, and graded with 0, 1, 2 and 3 points, respectively. The percentage of the visual field occupied by colored cells, < 5, 6% ~ 25, 26% ~ 50, 51% ~ 75 and > 75%, was graded with 0, 1, 2, 3 and 4 points, respectively. To calculate the final score, the two items were multiplied: 0 ~ 2 points (−), 3 ~ 4 points (+) 5 ~ 8 points (++) 9 ~ 12 points (+++). Two observers who are blinded read the results separately.

### Cell culture

The ovarian cancer cell lines (CAOV3, SKOV3, OVCAR3, and ES-2) were purchased from the Shanghai Cell Collection Center. The CAOV3, SKOV3, and OVCAR3 cells were cultured in conventional RPMI 1640 medium (BI, USA) containing 10% fetal bovine serum (BI, USA), whereas the ES-2 cells were cultured in McCoy’s 5A medium (BI, USA) containing 10% fetal bovine serum. The cells were incubated at 37 °C, 5% CO_2_ with saturated humidity.

### Cell transfection and construction of stably transfected cell lines

The ZNF703 small interfering (si) RNA was transfected into the CAOV3 and SKOV3 cell lines using the liposome method (Lipo 3000 transfection kit, GIBCO, Invitrogen). The cells were collected 48 h after transfection, and the effect of ZNF703 siRNA transfection on ZNF703 expression was determined using RT-PCR and western blotting. Within 48 h of transfection, the cells were used for functional experiments and flow cytometric analysis. The siRNA sequences of ZNF703 and HE4 and that of the negative control (GenePharma, Shanghai, China) are shown in Additional file 3: Table S1. The OVCAR3 and ES-2 cell lines were transfected using virus-mediated transfection and oncogene overexpression. The day before transfection, the cells were inoculated into 24-well cell culture plates at a density of 2500 cells/well. After overnight culture, RPMI 1640 culture medium, without serum, containing the lentivirus (MOI = 50) was added to the cells at 37 °C. After 8 ~ 12 h, complete medium containing serum was added.

### RT-PCR

The total RNA of the cells was extracted using Trizol according to the manufacturer’s instructions, and the purity and concentration of the RNA were determined using a UV spectrophotometer. The RT-PCR kit TAKARA047A (Takara Bio, Inc., Shiga, Japan) of the Super Script III First-Strand Synthesis System was used to reverse transcribe RNA into cDNA. The amplification conditions were: denaturation at 95 °C for 30 s, 95 °C for 5 s, and 60 °C for 30 s, for a total of 40 cycles. Real-time PCR amplification was performed using the 7500 Fast Real-Time PCR system. The primers used for ZNF703, PEA15, internal reference glyceraldehyde 3-phosphate dehydrogenase (GAPDH), and actin amplification are shown in Additional file 3: Table S2.

### Western blot

The RIPA cell lysis buffer was used to lyse the cells for 30 min at 4 °C, followed by centrifugation at 12000×g for 30 min at 4 °C. The supernatant was collected, and the protein concentration was determined using the BCA method. After denaturation, the protein was subjected to 10% sodium dodecyl sulphate-polyacrylamide gel electrophoresis (SDS-PAGE), and then transferred and blotted on a polyvinylidene difluoride (PVDF) membrane (Millipore, USA). The membrane was subsequently blocked for 2 h using 5% milk or bovine serum albumin (BSA) and incubated with the primary antibodies at 4 °C overnight. After washing with tris-buffered saline (TBST), the membrane was incubated with the secondary antibody (1:2000, Zhongshan Jinqiao, China) for 2 h and then washed with TBST. Proteins were visualized using the ECL reagent (Thermo Scientific ECL). The experiment was repeated three times. ECL (Thermo, USA) and the gel electrophoresis image analyzer GDS8000 were used for color development. The primary antibodies were shown in Additional file 3: Table S3.

### MTT cell proliferation assay

A total of 2000 cells per well were inoculated in a 96-well plate, and the cells were counted at 0 h and after adherence for 6 h, and 20 μL of 5 μg/mL MTT (5 mg/ml, Solarbio, Beijing, China) solution was added to each well. After incubation at 37 °C for 4 h, the medium was aspirated, 150 μl of dimethyl sulfoxide (DMSO) was added to each well, and after shaking for 5 min, the optical density (OD) values was determined.

### Apoptosis detection using flow cytometry

The Annexin-V-APC/7AAD (BD Biosciences, New York, USA) double staining method was used to detect apoptosis in ovarian cancer cell lines overexpressing ZNF703. Here, the Annexin-V-fluorescein isothiocyanate (FITC)/propidium iodide (PI) (KeyGen Biotech, Nanjing, China) double staining method to detect apoptosis when ZNF703 expression was inhibited by siRNA. The staining methods were carried out according to the instructions of the manufacturer.

### Cell cycle

Collect the cells in each group and fix with 70% ethanol was used to fix the cells overnight at 4 °C. A staining working solution of 500 μl PI/RNase (PI: RNaseA is prepared according to 9:1) (KeyGen Biotech, Nanjing, China) was added when the ethanol was removed using centrifugation in the following day. The cells were stained at 4 °C in the dark for 30 min. A cytometer (US BD company) was used for fluorescence detection.

### Wound healing test

Cells in the logarithmic growth phase were collected to prepare a single-cell suspension and seeded in 6-well plates. After the cell fusion reached 90%, the plate was gently scratched using a 100 μl pipette tip, then gently rinsed with PBS twice to replace the serum-free medium, and the width of the scratch was observed under a microscope. After incubation in serum-free medium for 24 h, the width of the scratch was again observed under a microscope.

### Invasion test

A total of 70 μl Matrigel glue (BD Corporation) was spread in the upper chamber of the Transwell chamber (Corning Coster) and placed in a 37 °C incubator to dry overnight. Then, 500 μl of medium containing 20% fetal bovine serum was added in the lower chamber, and 200 μl of the cell suspension (2 × 10^5^ cells) in serum-free medium was added in the upper chamber. After incubation at 37 °C for 48 h, the chamber was removed. The cells were fixed with 4% paraformaldehyde at room temperature for 30 min and stained with crystal violet for 30 min. The upper chamber surface was gently wiped clean with a cotton swab, and the number of tumor cells infiltrated by the lower chamber surface filter membrane was counted under a microscope.

### Co-immunoprecipitation

A total of 2 μg of anti-HE4 antibody (abcam: ab200828) or anti-rabbit IgG antibody (Zhongshan Jinqiao) was added to 500 μg protein lysate and incubated at 4 °C overnight. The next day, 40 μl protein A/G PLUS-Agarose beads (Santa Cruz: sc-2003) was added at 4 °C. After 6 h, the immunoprecipitated protein complex was added with 2 × loading buffer and boiled to denature the protein and separate it from the protein-G beads. The same method was performed using 2 μg of the anti-ZNF703 primary antibody to precipitate HE4, and the precipitated protein was detected using western blotting with the anti-HE4 primary antibody.

### Immunofluorescence and immunofluorescence co-localization

The cells on glass coverslips were fixed in 4% paraformaldehyde at room temperature for 20 min. After permeation with immunostaining permeation solution (Triton X-100, P0096, Beyotime) for 15 min, cells were blocked with PBS containing 5% BSA for 30 min. Subsequently, the cells were incubated with the primary antibody against ZNF703 (1:30) diluted in blocking buffer at 4 °C overnight and then incubated with Alexa Fluor 488-goat anti-mouse IgG (1:100) secondary antibody for 2 h. Next, the cells were rinsed with PBS for 15 min, and the nuclei were stained using 4′,6-diamidino-2-phenylindole dihydrochloride (DAPI) for 5 min. For detecting co-localization, the anti-HE4 primary antibody (1:200, DF8160, Affinity) and the anti-ZNF703 primary antibody (1:30, Santa Cruz) were incubated at the same time. Alexa Fluor 488-goat anti-mouse and Alexa Fluor 594-goat anti-mouse were used together as secondary antibodies.

### Nuclear cytoplasmic fractionation

Nuclear and cytoplasmic proteins were extracted and separated using the Nuclear and Cytoplasmic Protein Extraction Kit (Beyotime, Shanghai, China) according to the manufacturer’s instructions. The HE4 overexpression plasmid was constructed by GeneChem (Shanghai, China).

### Chromatin immunoprecipitation-sequencing (ChIP-seq)

The OVCAR3 cells were normally cultured in 10% FBS. Approximately 5 × 10^7^ cells were used in each ChIP-seq assay. After the cells were cross-linked, they were subjected to nuclear lysate extraction. Following sonication, monoclonal antibodies against ZNF703 were used for chromatin immunoprecipitation, followed by deep sequencing (ChIP-seq) (SeqHealth Tech, China). For ChIP-seq results, after obtaining the raw sequencing data (raw data), they were filtered, and high-quality sequencing data (clean data) were compared to the human genome (hg19). Then, the results were compared to the whole genome de novo peak calling, to study the protein’s binding preference in the genome, and to conduct a motif analysis of the binding site. Additional file 3: Table S4.

### ChIP-PCR

The ovarian cancer cells OVCAR3 were collected in the exponential growth phase and cross-linked in a medium containing 1% formaldehyde at room temperature for 15 min. Then, the cells were incubated with glycine at room temperature for 5 min to terminate cross-linking, followed by washing twice with PBS, and collection by centrifugation using a protease inhibitor in PBS. The rest of the procedure was performed according to the manufacturer’s instructions (SimpleChIP® Plus Enzymatic Chromatin IP Kit (Agarose Beads) #9004S, CST), using 10 μl of the anti-ZNF703 antibody (sc-271,896X, Santa Cruz) and 2 μg of the IgG antibody (5415S, [Mouse], CST). The primer used for the peak 150 corresponding to PEA15, is shown in Additional file 3: Table S5. The experiment was performed in two cell lines and repeated three times.

### Dual luciferase reporter gene assay

The plasmids employed in the luciferase experiments were synthesized by GeneChem (Shanghai, China). The PEA15 promoter fragments (2 kb upstream of TSS) were digested with restriction endonucleases KpnI and XhoI (Thermo Fisher Scientific). It recognizes the sequences GGTACC^CTCGAG. The enhancer-WT or enhancer-Mut sequences were digested with restriction endonucleases XbaI and XbaI. They recognize the sequences TCTAGA^TCTAGA. Then, they were ligated into the pGL3-basic plasmid (Promega). The HEK293T cells were divided into 24-well plates, co-transfected 500 ng each of the luciferase reporter gene construct of pGL3-Basic, PEA15 enhancer-wt, the mutant and the promoter region (2 kb upstream of TSS) plasmids with a total of 500 ng ZNF703 overexpression or control plasmid, and 2 μl transfection reagent lipo 3000 were added per well. Renilla luciferase plasmids was used as internal control transfected with 50 ng per well. After 48 h, the whole cell lysate was collected and used in the dual luciferase reporter assay (Promega, Madison, WI, USA), which was performed according to the manufacturer’s protocol (Promega). The data were calculated using Renilla luciferase as a control.

### Nude mouse xenograft model

Twelve 4-week-old female BALB/cA-nu nude mice, purchased from Beijing Huafukang Biosciences (Beijing, China), were maintained in specific pathogen-free conditions. Control vector/ZNF703 -overexpressed OVCAR3 cells (5 × 10^6^) cells were suspended in 150 μL of phosphate buffered saline (PBS) and injected subcutaneously into the axilla of mice (*n* = 6). The changes in tumor occurrence time, tumor formation rate, tumor formation number, tumor body diameter, mass and mouse body weight were recorded in each group every 4 days. The calculation method used for the tumor volume was V = 1/2 × a × b^2^ (a is the long axis and b is the short axis). All the mice were sacrificed after 37 days. The tumor samples were then fixed in 4% paraformaldehyde and embedded in paraffin. Continuous 4 μm-thick sections were cut and analyzed using hematoxylin and eosin (HE) or immunohistochemical staining. The animal study was approved by the Institutional Animal Research Committee of China Medical University.

### Bioinformation analysis

Functional and pathway enrichment analysis of genes co-expressed with ZNF703 were performed using DAVID (https://david.ncifcrf.gov), which integrates biological data and analysis tools to provide a systematic and comprehensive annotation of biological function. Genomic enrichment analysis was performed using the gene set enrichment analysis (GSEA) 3.0 software. The Oncomine database (http://www.oncomine.org), and GEPIA (http://gepia.cancer-pku.cn/), was used to analyze the mRNA expression level of PEA15 in the ovary.

### Statistical analysis

The data were counted using the *x*^*2*^ test and Fisher’s exact probability tests, and measurements of the data were performed using single factor analysis of variance. Statistical differences between two groups were carried out by using the t test, and one-way analysis of variance analysis was used for the comparison of more than two groups. A two-tailed *P* value of < 0.05 was considered statistically significant, *, *P* < 0.05; **, *P* < 0.01; ***, *P* < 0.001.

## Results

### Expression and clinical significance of ZNF703 in ovarian tissues

**Fig. 1:**
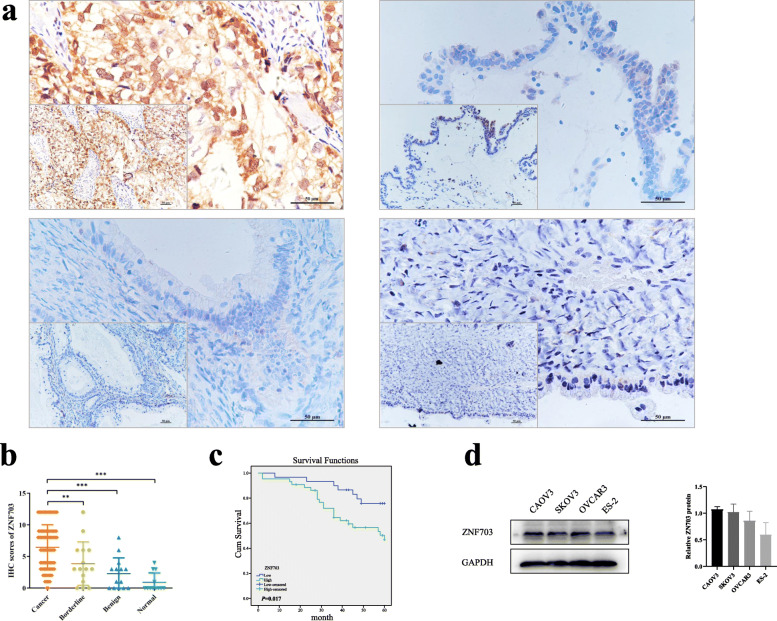
ZNF703 expression in clinical specimens and cell lines. **a** ZNF703 expression in ovarian tissues samples (Upper left: ovarian malignant tumor, upper right: ovarian borderline tumor, lower left: ovarian benign tumor, lower right: ovarian normal tissue) (× 400, lower left × 200). **b** Immunohistochemistry staining scores of ZNF703 in ovarian tissues samples. **c** Overall survival analysis according to ZNF703 expression in IHC (*P* = 0.017). **d** ZNF703 protein expression in four kinds of ovarian cell lines. For western blot, GAPDH was used as an internal control. The experiment was repeated three times. Data are presented as mean ± SD. *, *P* < 0.05; **, *P* < 0.01; ***, *P* < 0.001

To evaluate the expression of ZNF703 in ovarian cancer patients, we performed immunohistochemical staining of paraffin sections from clinical specimens. The results showed that ZNF703 was mainly expressed in the cell nucleus and cytoplasm of ovarian tissue (Fig. 1a). The positive and high-expression rates of the ovarian cancer group were 84.7% (83/98) and 60.2% (59/98), respectively, higher than those in the borderline group (66.7% [10/15] and 33.3% [5/15]; all *P* > 0.05), and significantly higher than those of the benign (50%[7/14] and 14.3%[2/14]; all *P* < 0.05) and normal (25%[3/12] and 0%[0/12]; *P* < 0.001) groups. In the ovarian borderline tumor group, the positive expression rate of ZNF703 was 66.7% (10/15), and the high expression rate was 33.3% (5/15), higher than that of the benign (50% [7/14] and 14.3% [2/ 14]) and normal groups (25% [3/12] and 0% [0/12]) (all *P* < 0.05) see Table 1. IHC scores was shown in Fig. 1b. A total of 98 samples of ovarian cancer were divided into the ZNF703 high-expression group (++/+++) and the ZNF703 low-expression group (−/+). The relationship between the expression of ZNF703 and clinicopathological parameters are shown in the Table 2. There was no significant correlation between ZNF703 expression and lymph node metastasis, clinical pathological stage or differentiation degree (*P* > 0.05). Follow-up of 98 patients with ovarian malignant tumors (as of April 30, 2019), and Kaplan-Meier survival analysis showed that the overall survival of ovarian cancer patients with high expression of ZNF703 was shorter than that of patients with low expression of ZNF703 (*P* = 0.017) (Fig. 1c).

**Table 1:** Expression of ZNF703 in different types of ovarian tissue

Group	Cases	Low		High		Positive rate(%)	High Positive rate(%)
		–	+	++	+++		
Malignant	98	15	24	28	31	84.7^a,b^	60.2^c,d^
Borderline	15	5	5	3	2	66.7^e,f^	33.3^g,h^
Benign	14	7	5	2	0	50	14.3
Normal	12	9	3	0	0	25	0

Note: a, malignant vs. benign (**, *P* = 0.006); b, malignant vs. normal (***, *P* < 0.001); c, malignant vs. benign (***, *P* < 0.001); d, malignant vs. normal (***, *P* < 0.001); e, borderline vs. benign (*P* = 0.362); f, borderline vs. normal (*, *P* = 0.031); g, borderline vs. benign (*P* = 0.39); h, borderline vs. normal (*, *P* = 0.047)

**Table 2:** Relationship between ZNF703 expression and clinicopathological parameters of ovarian epithelial malignant tumors

Groups	Cases	Low		High		Positive rate (%)	*P*-value	High expression rate (%)	*P*-value
		(−)	(+)	(++)	(+++)				
**FIGO stage**									
I-II	38	8	11	12	7	78.9%	*P* = 0.209	50.0%	*P* = 0.101
III-IV	60	7	13	16	24	85.5%		66.7%	
**Differentiation**									
Well- Moderate	49	9	14	14	12	81.6%	*P* = 0.4	53.1%	*P* = 0.149
Poor	49	6	10	14	19	87.8%		67.3%	
**Lymphatic metastasis**									
No	62	8	16	22	16	87.1%	*P* = 0.582	61.3%	*P* = 0.808
Yes	29	5	7	5	12	82.8%		58.6%	
Unknown^a^	7	2	1	1	3	71.4%		57.1%	
**Pathological type**									
Serous	45	6	11	13	15	86.7%	*P* = 0.158	62.2%	*P* = 0.440
Mucinous	9	3	1	2	3	66.7%		55.6%	
Endometrioid	15	3	4	4	4	80%		53.3%	
Clear cell carcinoma	7	2	3	1	1	71.4%		28.6%	
Poorly differentiated adenocarcinoma	22	1	6	5	10	95.5%		68.2%	

Note: a 7 patients without lymphadenectomy

Cox regression analysis was used to explore the relationship between different clinicopathological parameters and prognosis. Univariate analysis results showed that high expression of ZNF703, clinical analysis and lymph node metastasis were risk factors affecting the prognosis of ovarian cancer patients. Furthermore, the higher the expression of ZNF703, the worse was the prognosis (*P* < 0.05). Multivariate analysis results demonstrated that the clinical International Federation of Gynecology and Obstetrics (FIGO) stage was an independent risk factor for patient prognosis (Table 3). Taken together, these results indicate that the ZNF703 expression level was up-regulated in ovarian cancer tissues and was associated with poor prognosis.

**Table 3:** Univariate and Multivariate Cox Analysis of Different Clinicopathological Parameters with Ovarian Cancer

Variable	Categories	Univariate analysis		*P*	Multivariate analysis		*P*
		HR	95%CI		HR	95%CI	
Age	≤54	1.766	(0.815–3.830)	0.150			
	> 54						
Differentiation	Well-moderate	1.930	(0.895–4.163)	0.094			
	Poor						
FIGO stage	I-II	7.979	(2.397–26.554)	0.001^**^	7.399	(2.194–24.951)	0.001^**^
	III-IV						
Lymph node metastasis	NO	3.417	(1.506–7.753)	0.003^**^	1.475	(0.604–3.600)	0.394
	YES						
ZNF703	Low	2.716	(1.151–6.409)	0.022^*^	1.668	(0.641–4.338)	0.294
	High						

When analyzing the correlation of clinical specimens, it was found that ZNF703 had the correlation with HE4, in which Spearman correlation coefficient Rs = 0.213, *P* = 0.035 (Additional file 1: Table S1, Figure S1a).

### ZNF703 promotes ovarian cancer via inducing cell proliferation, cell cycle progression, and apoptosis inhibition

To study the effect of ZNF703 on the proliferation, cell cycle and apoptosis of ovarian cancer cells, its expression in ovarian cancer cell lines was investigated. The results showed that the expression of ZNF703 was slightly higher in the CAOV3 and SKOV3 cells and relatively lower in the OVCAR3 and ES-2 cells (Fig. 1d). When analyzing in cell line, we also verified the correlation between ZNF703 and HE4 at the protein and mRNA levels, and found that they were not significantly correlated (Spearman correlation coefficient: Rs = 0.552, *P* = 0.063; Rs = 0.510, *P* = 0.090) (Additional file 1: Figure S1b-e).

**Fig. 2:**
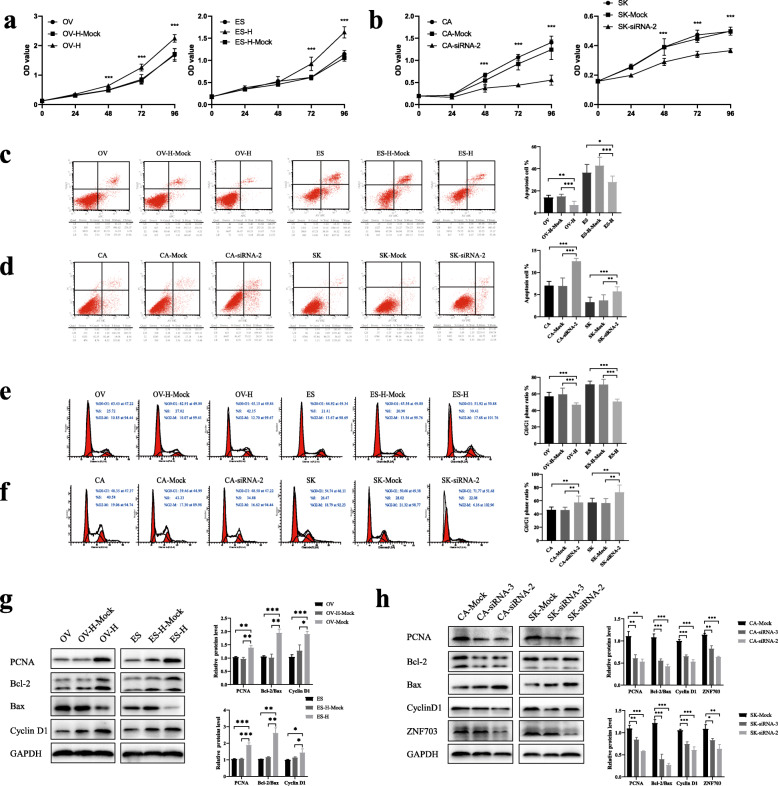
The influences of ZNF703 on proliferation and apoptosis and cell cycle in ovarian cancer cells. **a** Overexpression of ZNF703 promoted cell proliferation of ovarian cancer cells in MTT assay in OVCAR3 and ES-2 cell lines. **b** ZNF703-siRNA inhibited cell proliferation of ovarian cancer cells in MTT assay in CAOV3 and SKOV3 cell lines. **c** ZNF703 Overexpression decreased the cell apoptosis in OVCAR3 and ES-2 cell lines. **d** ZNF703-siRNA increased apoptosis of CAOV3 and SKOV3 cell lines. **e** Ovarian cells passed into S and G2/M phases after ZNF703 Overexpression. **f** G0/G1 phase arrest of ovarian cancer cells after ZNF703 siRNA transfection. **g** The increased proteins levels of PCNA, Bcl2/Bax, cyclin D1 were detected by western blot after overexpressed ZNF703. **h** Western blot analysis showed that the expression levels of PCNA, Bcl2/Bax, cyclin D1 proteins in ovarian cancer cells decreased after transfection with ZNF703-siRNA. The experiment was repeated three times. For western blot, GAPDH was used as an internal control. Data are presented as mean ± SD. *, *P* < 0.05; **, *P* < 0.01; ***, *P* < 0.001

The OVCAR3 and ES-2 cell lines were transfected using a lentivirus to overexpress ZNF703, whereas the CAOV3 and SKOV3 cell lines were transfected using the ZNF703 siRNA and the results were verified in protein level (Additional file 2: Figure S2a,b) and mRNA level (Additional file 2: Figure S2c,d). Enrichment analysis of the function of ZNF703 revealed that it was significantly associated with proliferation and apoptosis (Additional file 2: Figure S2e). The experimental results showed that ZNF703 overexpression significantly promoted the growth of OVCAR3 and ES-2 cells, especially at 48 h and 72 h (*P* < 0.05) (Fig. 2a). On the contrary, compared with the negative control, cell proliferation activity was inhibited (all *P* < 0.05) when the expression of the endogenous ZNF703 expression was reduced using siRNA (Fig. 2b). The western blot results showed the increased expression of proliferating cell nuclear antigen (PCNA) protein following ZNF703 overexpression and its decreased expression following ZNF703 siRNA knockdown (all *P* < 0.05) (Fig. 2g, h). Furthermore, flow cytometry results revealed that compared with the control group, the overall apoptosis of OVCAR3 and ES-2 cells was significantly reduced after ZNF703 overexpression (*P* < 0.05) (Fig. 2c), while apoptosis was significantly increased in CAOV3 and SKOV3 cells compared to the control group when ZNF703 expression was inhibited (Fig. 2d). Western blot detection of Bcl2/Bax changes also confirmed this result (Fig. 2g, h). In contrast, inhibition of ZNF703 induced cancer cell cycle arrest in the G0/G1 phase (all *P* < 0.05) (Fig. 2e), which was confirmed by the reduction of cyclin D1 protein (Fig. 2g, h). Overexpression of ZNF703 promoted the transition of the cell cycle from the G1 to S phase (Fig. 2f), and corresponding changes were also detected in the cyclin D1 levels (Fig. 2g, h). These findings indicated that ZNF703 might promote the proliferation of ovarian cancer cells and reduce apoptosis by accelerating the transition from the G0/G1 phase to S phase.

### ZNF703 promotes ovarian cancer cell invasion and migration in vitro

**Fig. 3:**
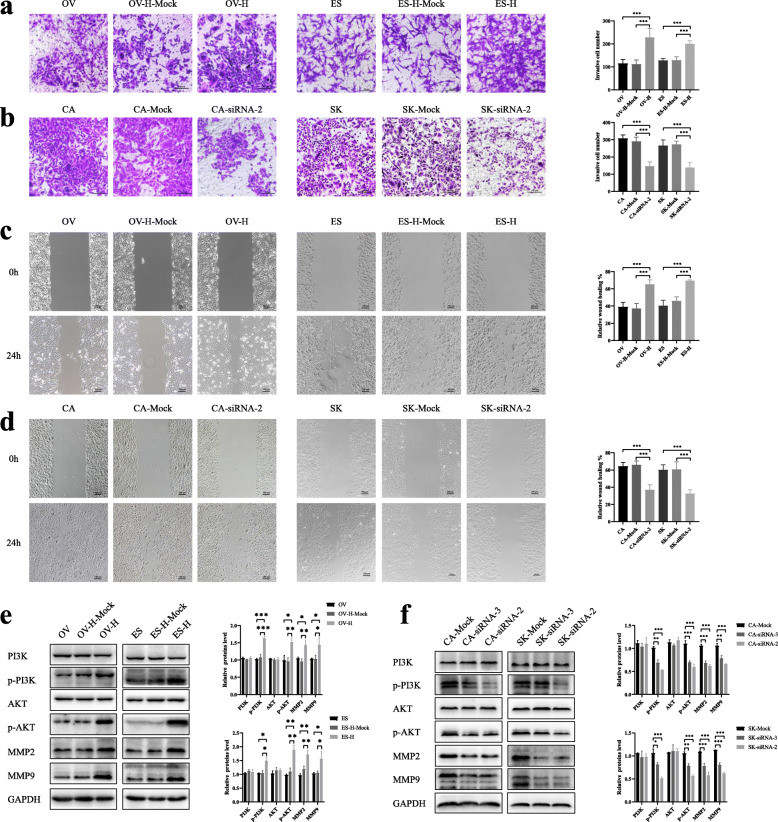
ZNF703 affected cell invasion and migration in ovarian cancer. **a** Overexpression of ZNF703 promoted cell invasion in OVCAR3 and ES-2 cell lines (×200). **b** ZNF703-siRNA suppressed invasion of CAOV3 and SKOV3 cell lines (×200). **c** ZNF703 overexpression promoted ovarian cancer cell migration in OVCAR3 and ES-2 cell lines (×100). **d** ZNF703-siRNA inhibited migration of CAOV3 and SKOV3 cell lines (×100). **e** Overexpression of ZNF703 enhanced the expression of p-PI3K, p-AKT, MMP2, MMP9. **f** Transfection of ZNF703 siRNA decreased the expression of p-PI3K, p-AKT, MMP2, MMP9. PI3K and AKT had no changes when ZNF703 was knockdown or overexpressed. The statistical results are on the right side of their corresponding positions. The experiment was repeated three times. GAPDH was used as an internal control. Data are presented as mean ± SD. *, *P* < 0.05; **, *P* < 0.01; ***, *P* < 0.001

To explore the effect of ZNF703 on the migration and invasion of ovarian cancer cells, invasion experiments and wound healing were performed. The results showed that ZNF703 overexpression significantly enhanced cell invasion (both *P* < 0.05) (Fig. 3a). On the other hand, inhibition of ZNF703 attenuated the cell invasion ability of the CAOV3 and SKOV3 cell lines (*P* < 0.05) (Fig. 3b). The experiment results showed that the wound healing speeds of ES-2 and OVCAR3 cells after ZNF703 overexpression were faster than those of the control group (both *P* < 0.05) (Fig. 3c). However, after inhibition of ZNF703 expression, the wound closure in CAOV3 and SKOV3 cells was slower (*P* < 0.05) (Fig. 3d). Furthermore, the western blotting results showed that the levels of matrix metalloproteinases 2 and 9 (MMP2 and MMP9) also increased when ZNF703 was overexpressed, which was consistent with the invasion results (*P* < 0.05) (Fig. 3e). At the same time, the opposite trend was observed in ovarian cancer cells in which ZNF703 expression was inhibited (Fig. 3f). These results indicate that ZNF703 promotes the migration and invasion of ovarian cancer cells.

### ZNF703 activates the PI3K/AKT pathway

It has been reported that PI3K and AKT are key factors regulating tumor progression [18]. This study also examined the effect of ZNF703 on the PI3K/ AKT signaling pathway and found that its overexpression increased the expression of phosphorylated PI3K (Tyr199) and AKT (Ser473), while its inhibition suppressed the expression and decreased the phosphorylation level of PI3K and AKT, whereas the expression levels of PI3K and Akt always remained unchanged (Fig. 3e, f).

### HE4 interacts with and promotes the nuclear translocation of ZNF703

**Fig. 4:**
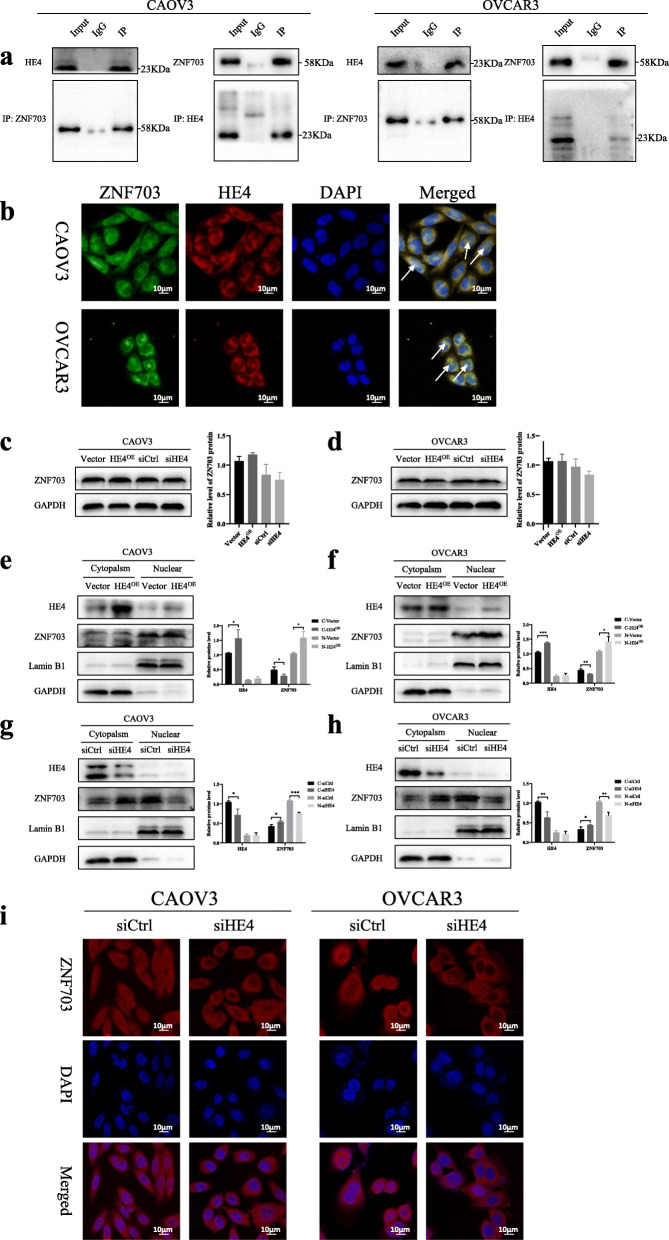
ZNF703 interacted with HE4 and HE4 promoted the nuclear translocation of the ZNF703. **a** The co-immunoprecipitation analysis of ZNF703 and HE4 was performed in ovarian cancer cells (The left two were performed in CAOV3 cell line and the right two were performed in OVCAR3 cell line. The corresponding molecular weight was added to the right side). **b** The immunofluorescence co-localization of ZNF703 and HE4 was performed in CAOV3 and OVCAR3 cells (The green color was for ZNF703 and the red color was for HE4, DAPI was blue, the colocalization was yellow) (×800). The arrow indicated the co-localization in the nucleus. **c, d** Western blot analysis of intracellular total ZNF703 protein in CAOV3 and OVCAR3 cells transfected with the overexpressed HE4 plasmid or control, or HE4 siRNA or control. **e, f** Western blot determined nuclear and cytoplasmic distribution of ZNF703 after nuclear cytoplasmic fractionation in CAOV3 and OVCAR3 cells transfected with the overexpressed HE4 plasmid or control. **g, h** Nuclear and cytoplasmic distribution of ZNF703 tested by western blot after nuclear cytoplasmic fractionation in CAOV3 and OVCAR3 cells transfected with HE4 siRNA or control. **i,** Immunofluorescent staining of ZNF703 in CAOV3 and OVCAR3 cells transfected with HE4 siRNA or control (× 800). The statistical results are on the right side of their corresponding positions. The experiment was repeated three times. Data are presented as mean ± SD. *, *P* < 0.05; **, *P* < 0.01; ***, *P* < 0.001

The expression of HE4, a glycoprotein, is increased in patients with ovarian cancer. We discovered ZNF703 while investigating HE4’s protein-protein interactions using the Y2H assay and performing a gene library screening to identify interactions between HE4 and interacting proteins in the early stage of ovarian cancer (unpublished data). To confirm the underlying mechanism, we performed co-immunoprecipitation (COIP), immunofluorescence colocalization, and nuclear and cytoplasmic separation analysis. The results of COIP showed that when ZNF703 was precipitated using an anti-HE4 antibody, it could be detected at a molecular weight of 58 kDa and vice versa (Fig. 4a). The results of immunofluorescence colocalization showed that ZNF703 and HE4 had a structural interaction (Fig. 4b). To study the role and underlying mechanism of HE4 and ZNF703 interactions in the development of ovarian cancer, we examined their subcellular distribution and localization in cells in which they were overexpressed or inhibited. Western blot analysis showed that the total protein expression of ZNF703 was not significantly affected by the differential expression of HE4 (Fig. 4c, d). Then, after overexpression or inhibition of HE4, nuclear and cytoplasmic separation analysis was carried out, and the cell distribution of ZNF703 in the CAVO3 and OVCAR3 cells was detected using western blotting. The results showed that overexpression of HE4 significantly increased the nuclear distribution of ZNF703 (Fig. 4e, f). In contrast, suppression of the expression of HE4 reduced the nuclear distribution of ZNF703 (Fig. 4g, h). Immunofluorescence results showed that a weaker ZNF703 expression signal was detected in the nucleus after transfection of the cells with HE4 siRNA, which could indicate that the upregulation of HE4 expression increases the nuclear translocation of ZNF703 (Fig. 4i).

Next, we tested the effects of HE4 in promoting the malignant biological behavior of ovarian cancer by ZNF703. We found that ZNF703 could restore the proliferation, invasion and migration functions inhibited by HE4 in ovarian cancer cells (Additional file 3: Figure S3a-c). But the knockdown of HE4 had no significant effects on the increased malignant biological ability induced by ZNF703 overexpression (Additional file 3: Figure S3a-c).

### Genome-wide characterization of ZNF703 transcriptional binding sites in ovarian cancer cells

**Fig. 5:**
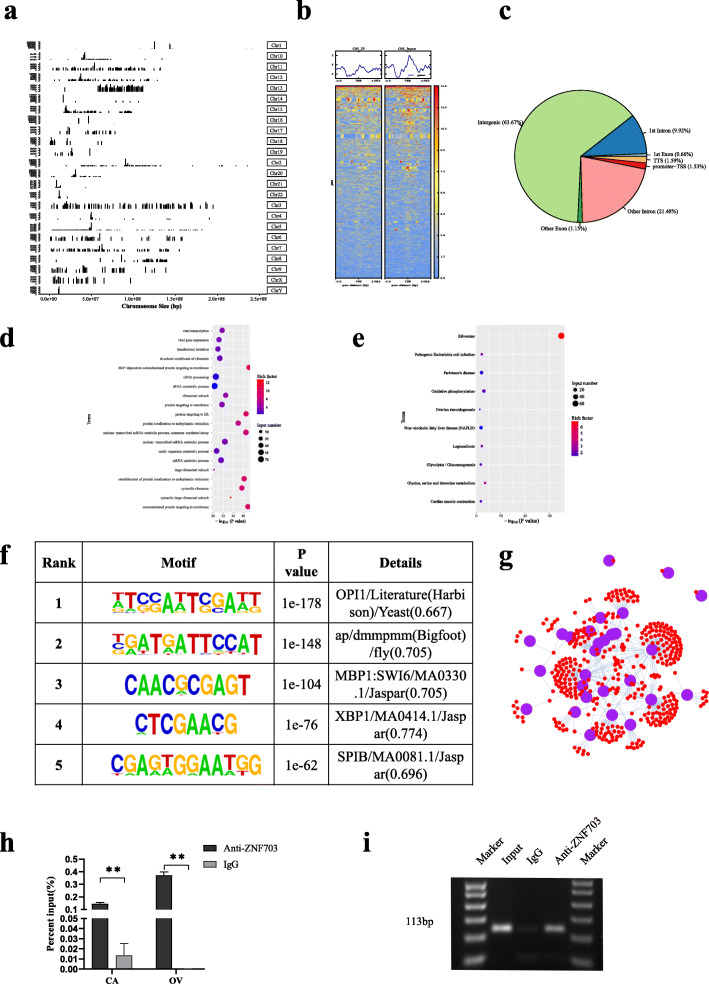
Identification of genome-wide DNA binding sites and transcription targets for ZNF703. **a** ChIP Peaks over chromosomes by ChIP-seq using the primary antibody against ZNF703 (The abscissa represents the length of the chromosome, the right represents the chromosome number, and the left ordinate represents the peak value of each chromosome). **b** The distribution of reads on both sides of the transcription start site (TSS)**. c** Pie diagram showed the ratios of ZNF703 binding sites located relative to a transcription unit including intergenic, 1st exon, 1st intron, TTS, promoter, other intron and other exon. **d** GO enrichment of Peak related genes (top 20 terms). **e** KEGG enrichment map of metabolic pathways of Peak-related genes (top 20 terms)**. f** Five common motifs with the most significant differences among peaks. **g** Interaction network diagram between TFs and genes based on sequencing results, purple represented the transcription factor or transcription factor family, red represented the target gene that TF may act through motifs. **h** ChIP-PCR to detect the binding of ZNF703 on the enhancer of PEA15 in CAOV3 and OVCAR3 cells using ZNF703 primary antibody, IgG was used as a negative control. The experiment was repeated three times. **i,** ChIP-PCR products were analyzed in OVCAR3 cell using horizontal agarose gel electrophoresis and visualized using UV. Data are presented as mean ± SD. *, *P* < 0.05; **, *P* < 0.01; ***, *P* < 0.001

ZNF703 is known to play a role as a carcinogenic TF in various tumors. However, the genomic binding pattern of ZNF703 in ovarian cancer remains unknown. In this study, the ChIP-seq was used to determine genome-wide target sites of ZNF703 in OVCAR3 ovarian cancer cells. The peaks over chromosomes showed different peak values, among them, there were the most enriched peaks on chromosome 3 and there were most high values of the peaks on chromosome 1 and 16.(Fig. 5a). A total of 1892 ChIP regions corresponding to 1314 unique RefSeq genes were identified (Fig. 5b). The ZNF703 binding sites located in the transcription termination site (TTS) and promoter-transcription start site (TSS) accounted for 3.12% of the total readings (Fig. 5c). Gene ontology (GO) analysis of the peak-related genes showed that the ZNF703 target genes were involved in various biological processes, such as metabolism, protein localization, and translation initiation (Fig. 5d). Kyoto Encyclopedia of Genes and Genomes (KEGG) analysis showed that the ZNF703 peak-related genes were significantly enriched in metabolism pathways including oxidative phosphorylation, glycine, serine, and threonine metabolism, and glycolysis/gluconeogenesis (Fig. 5e). The motifs shared between the peaks were scanned and the five motifs with the most significant differences were selected for display (Fig. 5f). The motifs could be combined with many TFs, and an interaction network diagram was drawn according to the correspondence between TFs and genes (Fig. 5g). PEA15 is an anti-apoptotic factor. The results of a previous study, showed the obvious effects of ZNF703 on the proliferation and apoptosis of ovarian cancer. In particular, this study found for the first time that ZNF703 binds to the enhancer region of PEA15 (Position: chromosome 1,160,199,305–160,199,593). ChIP-qPCR also confirmed that ZNF703 bound directly to the enhancer region of PEA15 in CAOV3 and OVCAR3 cells, and IgG was used as a negative control in the ChIP-qPCR verification (Fig. 5h, i).

### ZNF703 exerts oncogenic effects by directly promoting the transcription of PEA15

**Fig. 6:**
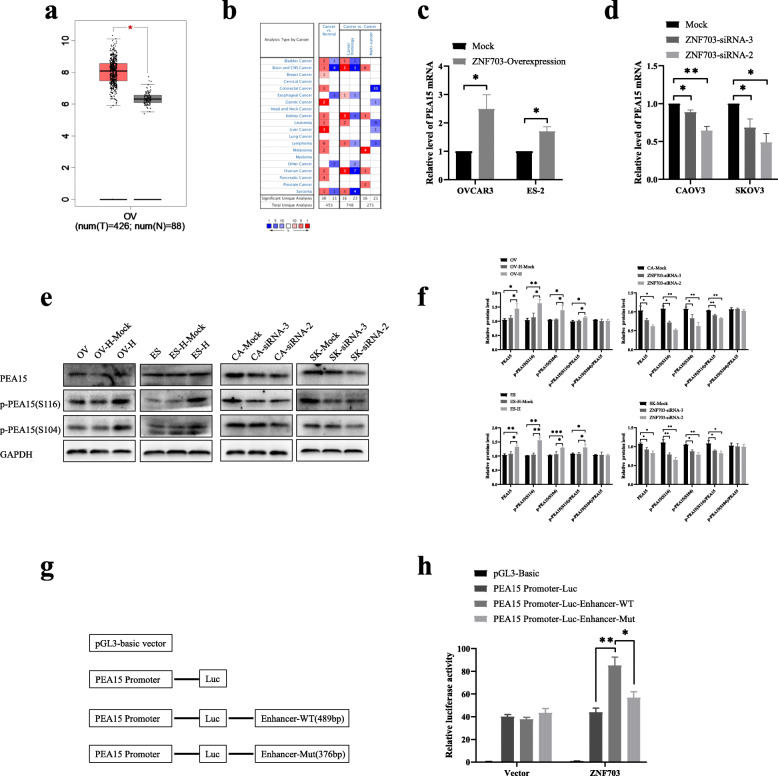
ZNF703 promoted the PEA15 expression epigenetically and phosphorylation at Ser116. **a, b** ZNF703 mRNA expression in the various tumors from GEPAI database and Oncomine database. **c, d** Validation of PEA15 mRNA levels changes using qRT-PCR analysis after upregulated or downregulated ZNF703 expression in cell lines (2^-ΔΔCт.^). **e** The PEA15 protein and phosphorylation levels changes in cell lines transfected with the overexpressed ZNF703 or control, or ZNF703 siRNA or control. **f** The relative gray value calculated for Fig. 6e. **g** Schematic diagram of dual luciferase reporter gene construction. **h** Dual luciferase activity assays were performed using firefly luciferase reporter vectors and Renilla luciferase served as an internal control. The experiment was repeated three times. Data are presented as mean ± SD. *, *P* < 0.05; **, *P* < 0.01; ***, *P* < 0.001

PEA15 was highly expressed in ovarian cancer in multiple databases (Fig. 6a, b). To understand the mechanisms underlying the effects of ZNF703 on PEA15, further experiments were conducted. The PCR results showed that after overexpressing ZNF703, the mRNA level of PEA15 increased (Fig. 6c), whereas after ZNF703 knockdown using ZNF703 siRNA, the levels of PEA15 decreased (Fig. 6d). Furthermore, the western blot results supported the above results at the protein level (Fig. 6e). In addition, phosphorylation levels of PEA15 were detected at Ser116 and Ser104, and it was found that p-PEA15 (Ser116), p-PEA15(Ser104) and p-PEA15(Ser116)/PEA15 increased after ZNF703 overexpression and decreased after its knockdown (Fig. 6e, f). Subsequently, we constructed a reporter gene vector with an enhancer fragment containing a region 100 bp upstream and downstream of peak 150 corresponding to the binding site, and deleted the binding site, verified using ChIP-PCR, to conduct a dual luciferase experiment (Fig. 6g). The results showed that ZNF703 overexpression enhanced the enhancer activity of PEA15 compared to the promoter activity of PEA15 alone, but when the binding site was deleted, the enhancer activity decreased (Fig. 6h). These results indicated that ZNF703 up-regulated the expression of PEA15 by directly binding to its enhancer region. Furthermore, ZNF703 also promoted the phosphorylation PEA15, particularly at Ser116 (Fig. 6f).

Meanwhile, we also detected the effect of HE4 on the expression of PEA15. PCR results showed that HE4 overexpression would increase the level of PEA15 mRNA, which decreased after knockdown (Additional file 4: Figure S4a). At the protein level, HE4 increases the protein expression of PEA15, but its effect on the phosphorylation of PEA15 is not significant (Additional file 4: Figure S4b, c).

Then, in clinical specimens, we discovered that PEA15 was highly expressed in ovarian cancer tissues (Additional file 5: Figure S5a, b, Table S2), and patients with PEA15 high expression had the poor survival prognosis (Additional file 5: Figure S5c). ZNF703 was related to the expression of PEA15 with the Spearman correlation coefficient Rs = 0.237 (*P* = 0.019) (Additional file 5: Figure S5d, Table S3), not only that, the expression of PEA15 was also related to HE4 (Spearman correlation coefficient Rs = 0.203, *P* = 0.045) (Additional file 5: Figure S5e, Table S4).

### Experiments in vivo demonstrate that ZNF703 promotes ovarian cancer cell proliferation

**Fig. 7:**
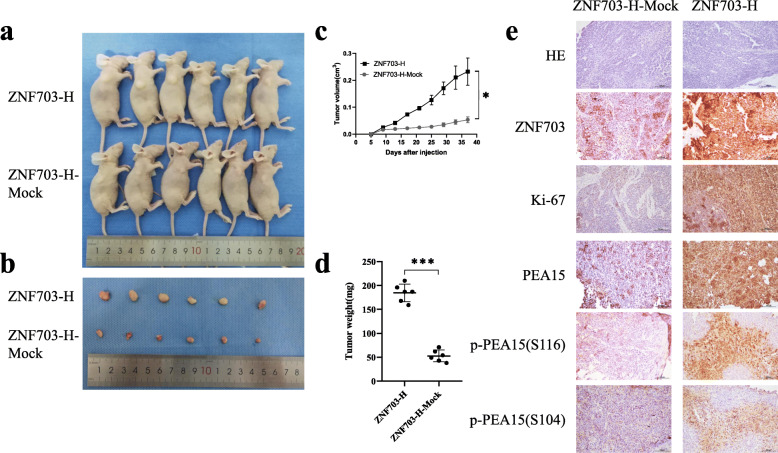
The impact of ZNF703 on tumor formation and proliferation ability in vivo. **a, b** Subcutaneous xenograft of nude mice model was performed using ZNF703 stable overexpression OVCAR3 cells. **c** The tumor volume were measured in all mice every 4 days (*P* = 0.011). **d** The tumor weight were measured in all mice at last. **e** Hematoxylin-Eosin staining and Immunohistochemistry staining on the samples of nude mouse xenograft tumors to display the expression of ZNF703, Ki-67, PEA15, p-PEA15(Ser116) and p-PEA15(Ser104) (× 200). The experiment was repeated three times. Data are presented as mean ± SD. *, *P* < 0.05; **, *P* < 0.01; ***, *P* < 0.001

To study the biological functions of ZNF703 in vivo, OVCAR3 cells stably overexpressing ZNF703 were injected subcutaneously into the axilla of nude mice (*n* = 6) (Fig. 7a, b). The overall tumor formation rate (100%, 6/6) was observed every 4 days. The results showed that the volume and weight of subcutaneous tumors in the ZNF703-overexpression group were significantly higher than those in the control group (Fig. 7c, d). Furthermore, in the ZNF703-overexpression group, the proliferation and invasion ability of cancer cells increased, and the cancer cells showed nodular growth. In addition, immunohistochemical staining of the tissue showed that compared with the control group, the color of the Ki-67 staining of cancer cells overexpressing ZNF703 was darker. Moreover, the tumor tissues of PEA15, p-PEA15 (Ser116) and p-PEA15 (Ser104) were stained more deeply in the ZNF703-overexpressing group than in the control group (Fig. 7e). These data indicate that ZNF703 significantly promotes the growth of cancer cells in vivo, is a key regulator of the proliferation of cancer cells in vivo and plays an important role in the malignant process of ovarian cancer.

## Discussion

Ovarian cancer is the most malignant gynecological tumor. It is often detected at an advanced stage with a short survival time and poor prognosis. ZNF703 is a TF belonging to the NET/NlZ family; it is located at 8p11.23 and is mainly distributed in the nucleus. Studies have demonstrated that ZNF703 and its homologs have six evolutionarily conserved domains, among which the three known domains are the spacer (SP) domain, the round head box (BTD) domain, and the cysteine2–histidine2 (C2H2) zinc finger domain [19]. Among the SP family proteins, these specific domains are necessary for the correct subcellular distribution and transcriptional inhibition of the NET/NlZ protein. The three newly discovered domains are unique to the NET protein family. Castro et al. [20], named these three specific domains as LP, PY, and YL based on the most abundant conserved amino acids in each of these domains. The NET protein lacks nuclear localization sequences and it is speculated that ZNF703 may interact with other proteins through the unique PY and YL domains to transport the NET protein into the nucleus [20]. This is consistent with the opinion that the C-terminal residue of the zebrafish Nlz1 protein was essential for the protein nuclear localization proposed by Runko et al. [21] and Sager strom et al. [22] Janesick A et al. [23] found that ZNF703 had different and separate functions in early mesoderm, neural crest, and substrate development. Other researchers have found that ZNF703, as a target of TFs Pax3 and Zic1, promoted the formation of the Xenopus neural crest [6]. Several studies have shown that the expression of ZNF703 was increased in malignant tumors and promoted the development of tumors [10, 24, 25]. In ovarian cancer, there is only one study showing that ZNF703 was upregulated and regulated the expression and role of LINC00460 in ovarian cancer tissues [26], but unfortunately we did not detect this binding during sequencing, which might be different from the cell lines or antibodies or kits we use, or even experimental deviations. This is worth thinking about.

Sircolomb et al. [27] found that ZNF703 inhibited the activity of transforming growth factor beta (TGFβ) receptor and the expression of E-cadherin and up-regulated the expression of pro-migratory P120 catenin, thereby promoting cell proliferation and migration and reducing intercellular adhesion. Furthermore, ZNF703-overexpressing cells altered retinoblastoma-associated protein (RB1) phosphorylation and downregulated P27kip1 protein, thereby up-regulating the expression of E2F1 and cyclin E1 (CCNE1) (G1/S-specific cyclin E1), inducing cells to escape from the R restriction point of the G1 phase and enter S phase [27]. This study found that ZNF703 was overexpressed in ovarian cancer, and the survival time of patients with high expression of ZNF703 was significantly shortened. But ZNF703 was not an independent prognostic factor. In the cells overexpressing ZNF703, it was found that their proliferation was accelerated and apoptosis was reduced; thus, more cells entered the S and G2/M phases. However, when siRNA was used to down-regulate the expression of ZNF703 in the ovarian cancer cell lines, cell proliferation slowed down and apoptosis increased; moreover, cell cycle arrest at the G1 phase was observed. Regarding cell invasion and migration, the ability of ovarian cancer cells to invade and migrate decreased after the expression of ZNF703 was inhibited, and increased after ZNF703 overexpression. In vivo experiments also proved that ZNF703 overexpression promoted tumor formation and growth. This was consistent with the reported role of ZNF703 as an oncogene [10, 28, 29]. As the PI3K/AKT pathway is a key coordinator of intracellular signal transduction that regulates multiple cellular processes [18], it is often excessively activated in human malignancies and plays a key role in cancer progression [30]. The results in ovarian cancer cell lines also confirmed that ZNF703 could activate the PI3K/AKT pathway to promote the malignant biological behaviors of ovarian cancer cells. In fact, ZNF703 is also a protein with phosphoserine at position 252. We guessed whether it was involved in the phosphorylation process of PI3K/AKT so as to directly participate in its activation. On the other hand, we speculated whether ZNF703 regulated the upstream activators of PI3K/AKT pathway, such as RTKs, FAK, JAK [31]. Considering that ZNF703 is a transcription factor, whether it regulated the transcription of PI3K/AKT pathway or its upstream, although this was not found in our results until now.

We discovered ZNF703 by yeast two-hybrid of HE4. Subsequent verification experiments also confirmed that there was an interaction between ZNF703 and HE4, but the functional impact of HE4 on ZNF703 remains unknown. On the one hand, Castro et al. [20] transfected the human HEK293T cell line using an overexpression vector encoding MYC markers, and proved that ZNF703/NLZ1 was mainly located in the nucleus, however the NLS (nuclear transfer mechanism) pathway of ZNF703 was not identified. Furthermore, it has been found that the central ZNF703 PY domain and the C-terminal YL domain were very important for the nuclear localization of proteins [20]. This conclusion was similar to that proposed by other researchers studied in zebrafish [21, 22]. Therefore, it was speculated that ZNF703 may associate via its LY and PY domains with other proteins to participate in a known nuclear localization pathway and enter the nucleus. On the other hand, HE4 is a glycoprotein that has been used clinically as a tumor marker for ovarian cancer. Many studies have focused on the role of HE4 as a secreted protein, but one study reported that HE4 could interact with importin-4 for nuclear translocation [32]. Based on the current research conclusions, it is speculated that ZNF703 and HE4 may interact through the LY and PY domains of ZNF703, and that HE4 may promote the nuclear translocation of ZNF703. Furthermore, it has been found that after inhibiting the expression of HE4, ZNF703 accumulated in the cytoplasm. Moreover, when HE4 was overexpressed, the nuclear expression of ZNF703 increased, but this was not obvious in the immunofluorescence images, probably because ZNF703 was already highly expressed in the nucleus, irrespective of the levels of HE4, making it difficult to detect changes in fluorescence intensity. However, whether ZNF703 and HE4 interact via the LY and PY domains, and whether the nuclear translocation of ZNF703 depends on the interaction between the two remains to be confirmed. This is one of our future research aims. So we speculated that ZNF703 could interact with HE4 in the cytoplasm and ZNF703 could be brought into the nucleus by HE4, or with the help of HE4, when the nuclear pore is opened. The correlation between HE4 and ZNF703 was not significant in cell lines, but it was significant in clinical specimens, although the correlation coefficient was very low. This might be because HE4 was highly expressed in cell lines, but for clinical patients, the level of HE4 was quite different. But it also showed that the correlation between the two was not high.

In recent years, with the development of gene sequencing technology, genomic analysis has provided the prospect of revealing the characteristics of tumors, and an opportunity to explore the underlying mechanism of ovarian cancer development. At present, there is little evidence about the transcription function and epigenetic regulation of ZNF703. Nakamura et al. [5] demonstrated that ZNF703 could suppress gene expression by recruiting the histone deacetylation complex (HDAC) and regulate the embryonic development process, such as the differentiation of vertebrate hindbrain into rhomboids. Sircoulomb et al. [27] proposed that ZNF703 inhibited the transcription of related genes by forming a nuclear transcription complex with DNA damage-binding protein 1 (DDB1) and cullin 4 (CUL4) associated factor 7 (DCAF7), CUL4, prohibitin-2 (PHB2), and nuclear receptor corepressor 2 (NCOR2) and exerted its regulatory effects on cell proliferation, differentiation, apoptosis, and cell cycle. In terms of promoter binding, Holland et al. [29] claimed that ZNF703 overexpression can lead to ZNF703 binding to the promoter site of the TGFβ receptor II (TGFβR2), preventing TGFβ from inhibiting cell proliferation. ChIP-sequencing technology was used to detect the binding of ZNF703 on chromatin in ovarian cancer cells. This study was the first to conduct epigenetic studies on ZNF703 in vitro. However, whether the transcriptional activity of TFs differs in different tissues, and whether it is related to the formation of transcription complexes with other proteins and the target genes that are bound, should be further investigated. Furthermore, whether the binding of ZNF703 to chromatin is related to the structure of ZNF703 requires further study.

An inhibitor of apoptosis (IAP) family member, PEA15 was cloned from astrocytes [33]. It is a small molecule protein with a broad-spectrum anti-apoptotic function. Studies have shown that p-PEA15(Ser116) can bind to other proteins through its DED region, thereby inhibiting the formation of the death-inducing complex (DISC), inactivating caspase-8 and caspase-10 and preventing the activation of the caspase cascade. This results in the inhibition of apoptosis in tumor cells, and the occurrence, development and metastasis of tumors [15–17, 34, 35]. PEA15 has two phosphorylation sites, Ser104 and Ser116. Ser104 can be phosphorylated by protein kinase C (PKC) [36], while Ser116 can be phosphorylated by calmodulin-dependent protein kinase II (CaM kinase II) or AKT [37, 38]. Phosphorylation at Ser116 enhances the binding of Fas-related proteins to the death domain (FADD) and to caspase-8, inhibiting apoptosis [36, 37]. PEA15 appears to play a role in promoting or suppressing tumors. For example, the expression of PEA15 was found to be increased in non-small cell lung cancer [39], chronic lymphocytic leukemia [40], thyroid cancer [41], prostate cancer [42] and liver cancer cells [43], where it inhibited apoptosis and promoted tumor growth. In contrast, increased PEA15 expression was also found to inhibit extracellular signal-regulated kinase (ERK)-dependent functional transcription and proliferation in ovarian cancer [44] and breast cancer cells [45]. But there is also evidences in ovarian cancer that PEA15 was positively correlated with the FIGO stage and cell proliferation was obstructed by knockdown of PEA15 [46].

We also found that HE4 affected the transcription and protein expression levels of PEA15, but had no effect on the phosphorylation of PEA15, and PEA15 had an anti-apoptotic effect after phosphorylation. This was also consistent with our observation of the effects of HE4 knockdown on ZNF703 roles. It showed that HE4 affected the nuclear translocation of ZNF703, but it was not enough to cause significant changes in the functions of ZNF703. Of course, we only tested these biological behaviors. As for other aspects, such as metabolism, chemotherapy resistance, angiogenesis, etc., this will be our next research direction.

The results of this study demonstrated that ZNF703 promotes the expression of PEA15 by binding to its enhancer region. Furthermore, ZNF703 played a significant role in promoting cancer gene expression, but this seemed inconsistent with previous studies in which PEA15 was found to inhibit ovarian cancer cell proliferation. Moreover, this study determined the protein expression levels of PEA15, p-PEA15(Ser116) and p-PEA15(Ser104). The results showed that after overexpression of ZNF703, the phosphorylation of p-PEA15(Ser116)/PEA15 increased, consistent with the increased phosphorylation level of AKT found in this study. The results of Lee J et al. [47] also confirmed that PEA15 in ovarian cancer mostly exists in the phosphorylated form. Fiory F et al. [14] also suggested that some differences in PEA15-mediated effects may depend on the phosphorylation status or cellular environment, which might be important in determining whether PEA15 regulates cell survival or apoptosis.

## Conclusions

In conclusion, this is the first study to describe the role and underlying mechanism of ZNF703’s oncogenic action in the development of ovarian cancer. ZNF703 promoted the malignant behavior of ovarian cancer cells through the PI3K-AKT pathway. We also demonstrated that HE4 interacted with ZNF703, and promoted its nuclear translocation. Furthermore, ZNF703 could bind to the enhancer region of PEA15 to promote its transcription. Moreover, we also considered other potential functions of HE4 except that of a secreted protein. In the future, ZNF703 may be used as a prognostic factor and trigger new research ideas for further understanding the underlying pathogenesis and improving the diagnosis and treatment of ovarian cancer.

## Supplementary Information

**Additional file 1:** **Table S1** The correlation between ZNF703 and HE4 expression in ovarian cancer. **Figure S1.** The correlation between ZNF703 and HE4 expression in ovarian cancer both in clinical specimens and cell lines. a The correlation between ZNF703 and HE4 expression with Scatter plot in in clinical specimens. b The protein expression levels of ZNF703 and HE4 in four ovarian cancer cell lines. c The correlation between ZNF703 and HE4 protein expression levels with Scatter plot in in cell lines. d The mRNA expression levels of ZNF703 and HE4 in four ovarian cancer cell lines (ΔCт). e The correlation between ZNF703 and HE4 mRNA expression levels with Scatter plot in in cell lines (ΔCт). Data are presented as mean ± SD. *, *P* < 0.05; **, *P* < 0.01; ***, *P* < 0.001.

**Additional file 2:** **Figure S2.** The verification of ZNF703 siRNA or lentivirus transfection in ovarian cell lines and functional enrichment analysis. a Western blot showing the protein levels of ZNF703 in OVCAR and ES-2 cells after overexpressing. b ZNF703 siRNA confirmation in CAOV3 and SKOV3 cells by western blot. c qRT-PCR for ZNF703 mRNA after overexpressing (2-^ΔΔCт^). d Analysis of ZNF703 mRNA in cells transfected with siRNA by qRT-PCR (2-^ΔΔCт^). e The bubble plot of top 20 biological functions and pathways related to ZNF703. Data are presented as mean ± SD. *, *P* < 0.05; **, *P* < 0.01; ***, *P* < 0.001.

**Additional file 3:** **Figure S3**. HE4 impacts on the functional activity of ZNF703. a MTT assays showed HE4 knockdown inhibited cell growth of OVCAR3 and ES-2 cells, while ZNF703 overexpression could restore promoted cell growth. b. HE4 knockdown inhibited cell migration in OVCAR3 and ES-2 cell lines, which could be restored by overexpressed ZNF703 (× 100). c. HE4 knockdown inhibited cell invasion in OVCAR3 and ES-2 cell lines, which could be restored by overexpressed ZNF703 (× 200). Data are presented as mean ± SD. *, *P* < 0.05; **, *P* < 0.01; ***, *P* < 0.001.

**Additional file 4:** **Figure S4**.The effects of HE4 on the ZNF703-dependent regulation of PEA15. a The PEA15 mRNA levels after HE4 siRNA knockdown or overexpression in OVCAR3 and ES-2 cells by qRT-PCR (2-^ΔΔCт^). **b** The PEA15 protein levels after HE4 siRNA knockdown with or without ZNF703 overexpression in OVCAR3 and ES-2 cells by western blot. **c** The PEA15 protein levels after HE4 overexpression with or without ZNF703 siRNA knockdown in OVCAR3 and ES-2 cells by western blot. Data are presented as mean ± SD. *, *P* < 0.05; **, *P* < 0.01; ***, *P* < 0.001.

**Additional file 5:** **Figure S5**.The expression of PEA15 in clinical specimens and the correlation with ZNF703 or HE4. a PEA15 expression in ovarian tissues samples (Upper left: ovarian malignant tumor, upper right: ovarian borderline tumor, lower left: ovarian benign tumor, lower right: ovarian normal tissue) (× 400, upper right × 200). b Immunohistochemistry staining scores of PEA15 in ovarian tissues samples. c Overall survival analysis according to PEA15 expression in IHC (*P* = 0.032). d The correlation between ZNF703 and PEA15 expression with Scatter plot in in clinical specimens. e The correlation between PEA15 and HE4 expression with Scatter plot in in clinical specimens. Data are presented as mean ± SD. *, *P* < 0.05; **, *P* < 0.01; ***, *P* < 0.001. **Table S2** Expression of PEA15 in different types of ovarian tissue. **Table S3** The correlation between ZNF703 and PEA15 expression in ovarian cancer. **Table S4** The correlation between PEA15 and HE4 expression in ovarian cancer.

**Additional file 6:** **Figure S6**. The effects of overexpression ZNF703 after ZNF703 siRNA knockdown on cell proliferation in cell lines. **a** MTT assays showed that the overexpression of ZNF703 could rescue the inhibitory effects of ZNF703 knockdown on cell proliferation in CAOV3 cells. **b** MTT assays showed that the overexpression of ZNF703 could rescue the inhibitory effects of ZNF703 knockdown on cell proliferation in SKOV3 cells. Data are presented as mean ± SD. *, *P* < 0.05; **, *P* < 0.01; ***, *P* < 0.001.

**Additional file 7:** **Figure S7**. The interaction of ZNF703 and HE4 during HE4 siRNA knockdown. **a** The immunofluorescence co-localization of ZNF703 and HE4 was performed in CAOV3 and OVCAR3 cells transfected with HE4 siRNA or control (The green color was for ZNF703 and the red color was for HE4, DAPI was blue, the colocalization was yellow)(× 800). The arrow indicated the co-localization in the nucleus. b The co-immunoprecipitation analysis of ZNF703 and HE4 was performed in ovarian cancer cells transfected with HE4 siRNA (The upper two were performed in CAOV3 cell line and the lower two were performed in OVCAR3 cell line. The corresponding molecular weight was added to the right side).

**Additional file 8:** **Table S5.** ZNF703 binding DNA sites (Displaying some of results including PEA15). **Table S6.** Primers for qRT-PCR (5′ to 3′). **Table S7.** siRNA sequences (GenePharma, Shanghai, China). **Table S8.** Primers for ChIP-PCR (5′ to 3′). **Table S9.** Antibodies used in the manuscript. **Table S10.** Reagents and Kits used in this work.

**Additional file 9.** : The details regarding the plasmids employed in the luciferase experiments.

**Additional file 10.** : Original Figs. 5a-e with each individual.

## Data Availability

The ChIP-seq raw data for ZNF703 from this study have been deposited in NCBI Sequence Read Archive with accession number PRJNA611869. Other datasets used and/or analysed during the current study are available from the corresponding author on reasonable request.

## Abbreviations

ZNF703: Zinc finger protein 703
ChIP-seq: Chromatin immunoprecipitation sequence
COIP: Co-immunoprecipitation
TFs: Transcription factors
NLS: A nuclear localization sequence
TSS: Transcription Start Site
PCNA: Proliferating cell nuclear antigen
KEGG: Kyoto Encyclopedia of Genes and Genomes
GO analysis: Gene ontology analysis
CCNE1: G1/S-specific cyclin E1
DDB1: DNA damage-binding protein 1
DCAF7: DNA damage-binding protein 1 and cullin 4 associated factor 7
PHB2: Prohibitin-2
NCOR2: Nuclear receptor corepressor 2
TGFβR2: TGFβ receptor II
