# Supplementary material for: ZNF703 promotes tumor progression in ovarian cancer by interacting with HE4 and epigenetically regulating PEA15
Source: J Exp Clin Cancer Res. 2020 Nov 27;39:264. doi: 10.1186/s13046-020-01770-0 (PMC7693506; doi:10.1186/s13046-020-01770-0)
Supplement: Supplementary file 1 — Additional file 1: Table S1 The correlation between ZNF703 and HE4 expression in ovarian cancer. Figure S1. The correlation between ZNF703 and HE4 expression in ovarian cancer both in clinical specimens and cell lines. a The correlation between ZNF703 and HE4 expression with Scatter plot in in clinical specimens. b The protein expression levels of ZNF703 and HE4 in four ovarian cancer cell lines. c The correlation between ZNF703 and HE4 protein expression levels with Scatter plot in in cell lines. d The mRNA expression levels of ZNF703 and HE4 in four ovarian cancer cell lines (ΔCт). e The correlation between ZNF703 and HE4 mRNA expression levels with Scatter plot in in cell lines (ΔCт). Data are presented as mean ± SD. *, P < 0.05; **, P < 0.01; ***, P < 0.001. [file 13046_2020_1770_MOESM1_ESM.pdf]

Table S1 The correlation between ZNF703 and HE4 expression in ovarian cancer (n=98)

ZNF703	HE4				case
	-	+	++	+++	
-	5	2	4	4	15
+	4	7	2	11	24
++	2	3	10	13	28
+++	3	6	2	20	31
**case**	14	18	18	48	98

a

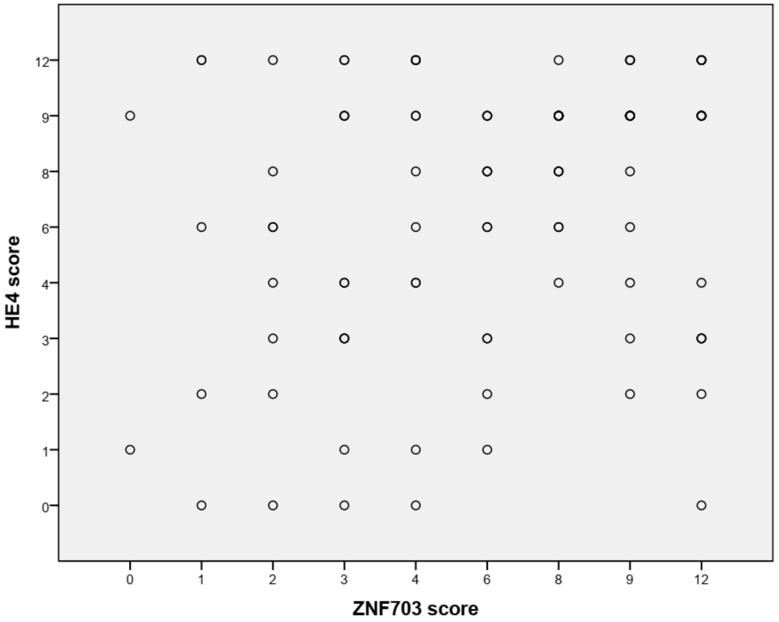

Spearman correlation coefficient Rs=0.213, *P*=0.035

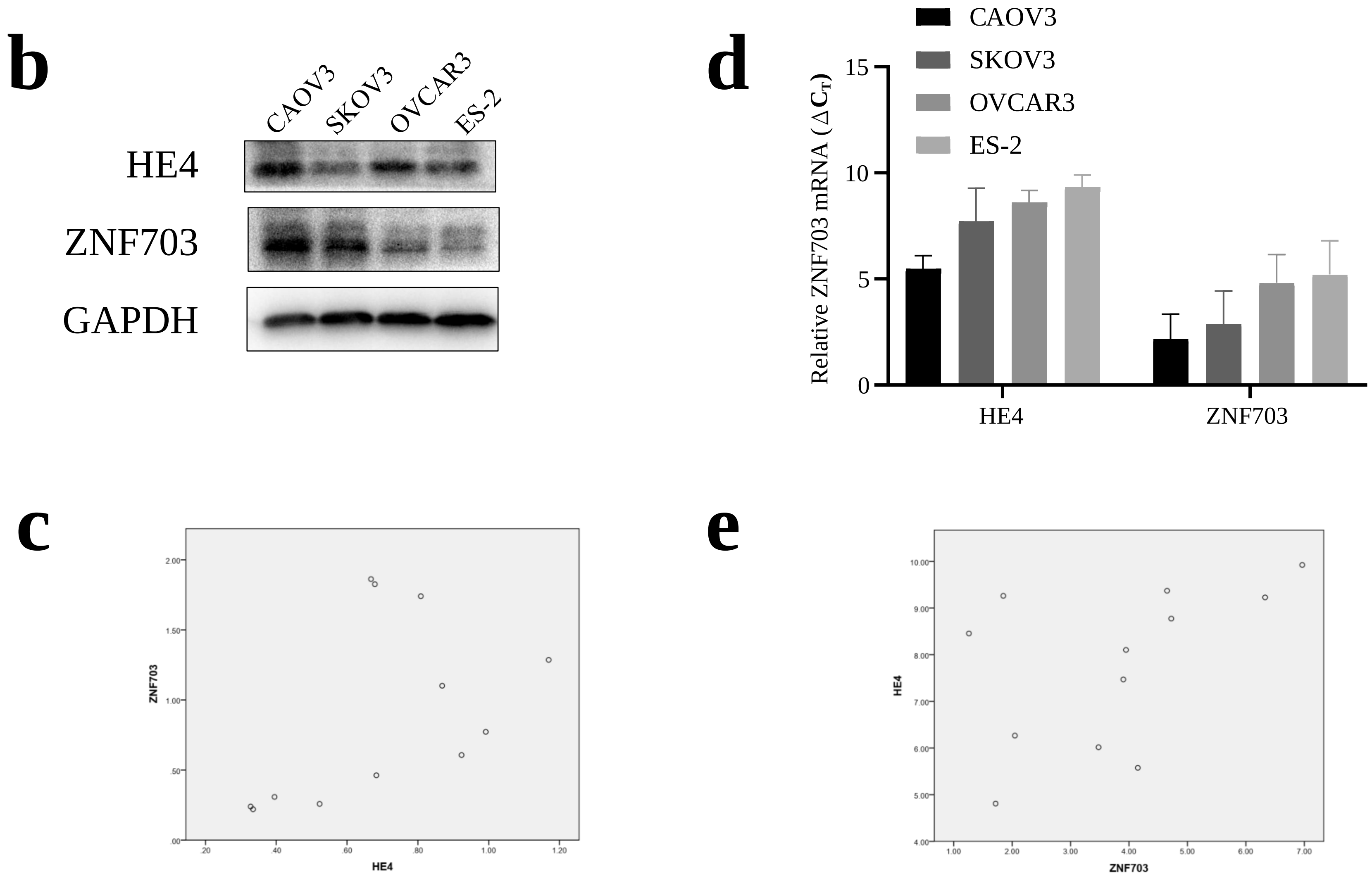

Spearman correlation coefficient Rs=0.552, *P*=0.063

Spearman correlation coefficient Rs=0.510, *P*=0.090
