# Supplementary figures and images for: ZNF703 promotes tumor progression in ovarian cancer by interacting with HE4 and epigenetically regulating PEA15
Source: J Exp Clin Cancer Res. 2020 Nov 27;39:264. doi: 10.1186/s13046-020-01770-0 (PMC7693506; doi:10.1186/s13046-020-01770-0)

**a**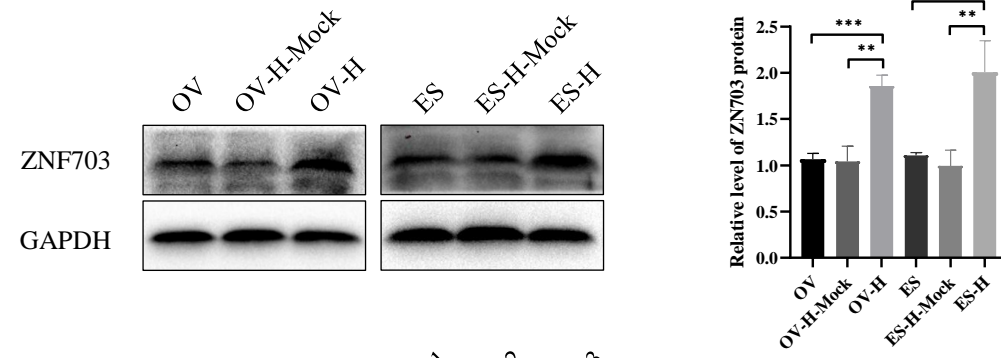**b**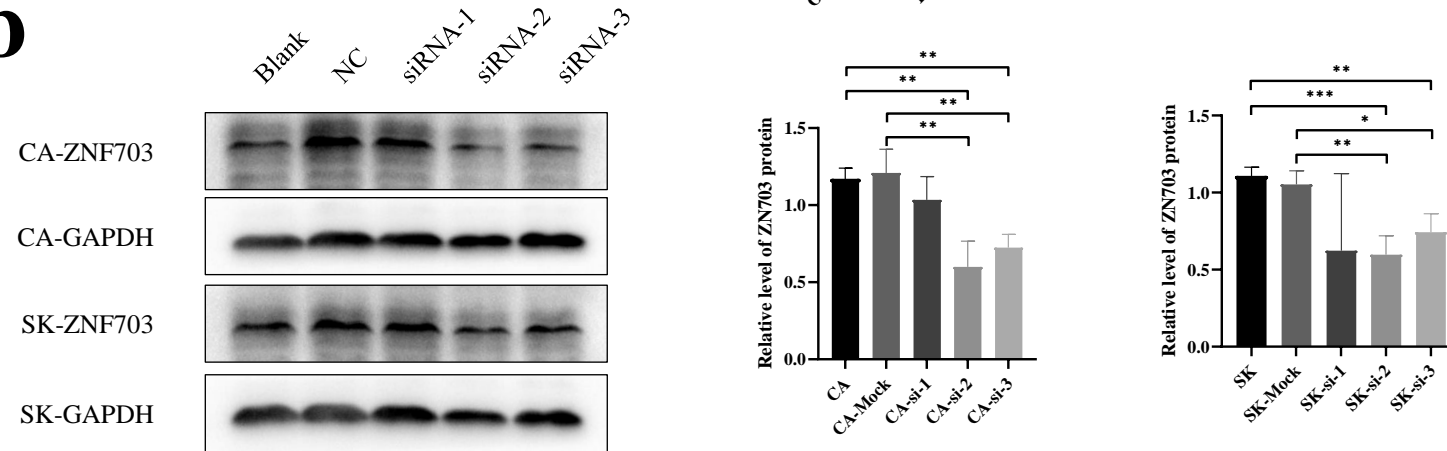**c**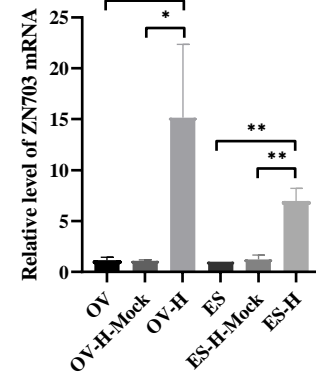**d**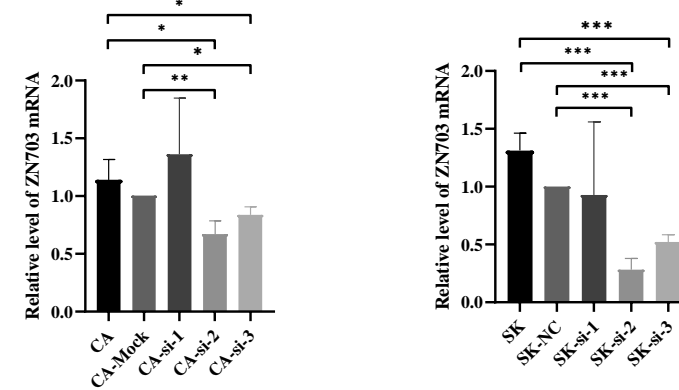**e**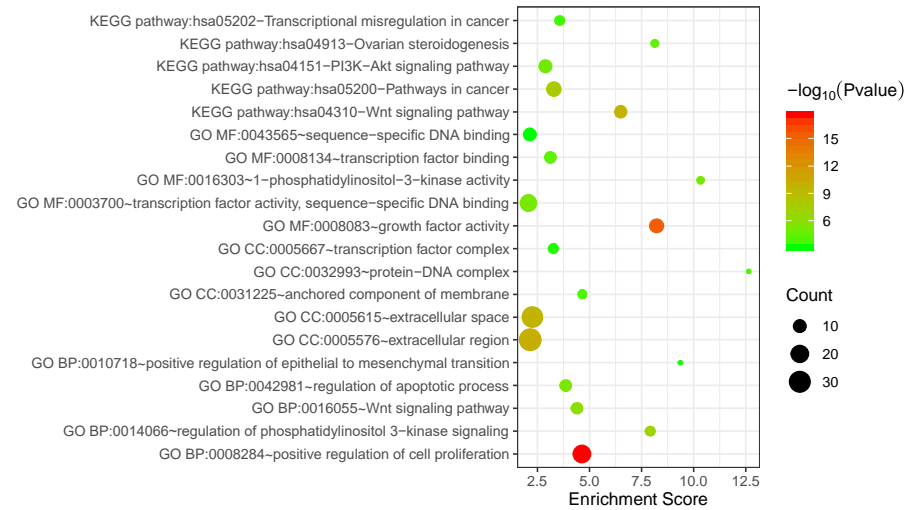

Supplement: Supplementary file 2 — Additional file 2: Figure S2. The verification of ZNF703 siRNA or lentivirus transfection in ovarian cell lines and functional enrichment analysis. a Western blot showing the protein levels of ZNF703 in OVCAR and ES-2 cells after overexpressing. b ZNF703 siRNA confirmation in CAOV3 and SKOV3 cells by western blot. c qRT-PCR for ZNF703 mRNA after overexpressing (2-ΔΔCт). d Analysis of ZNF703 mRNA in cells transfected with siRNA by qRT-PCR (2-ΔΔCт). e The bubble plot of top 20 biological functions and pathways related to ZNF703. Data are presented as mean ± SD. *, P < 0.05; **, P < 0.01; ***, P < 0.001. [file 13046_2020_1770_MOESM2_ESM.pdf]

**a**

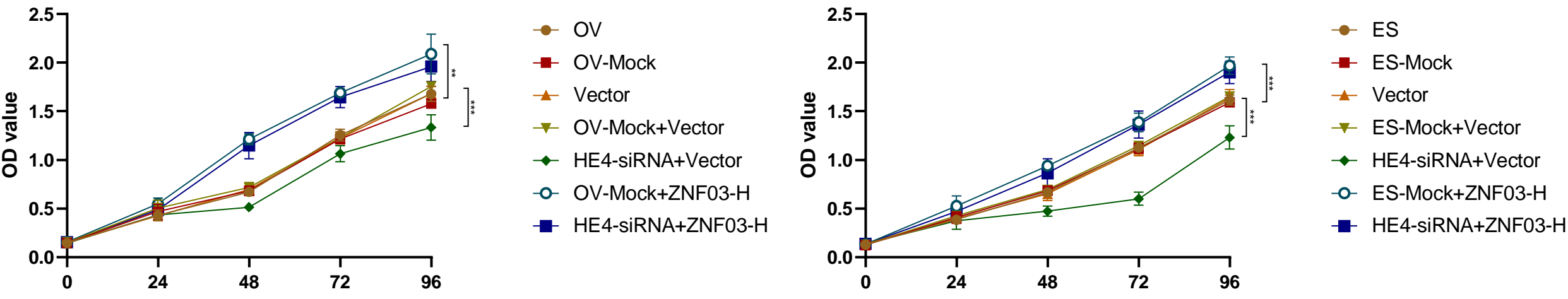

**b**

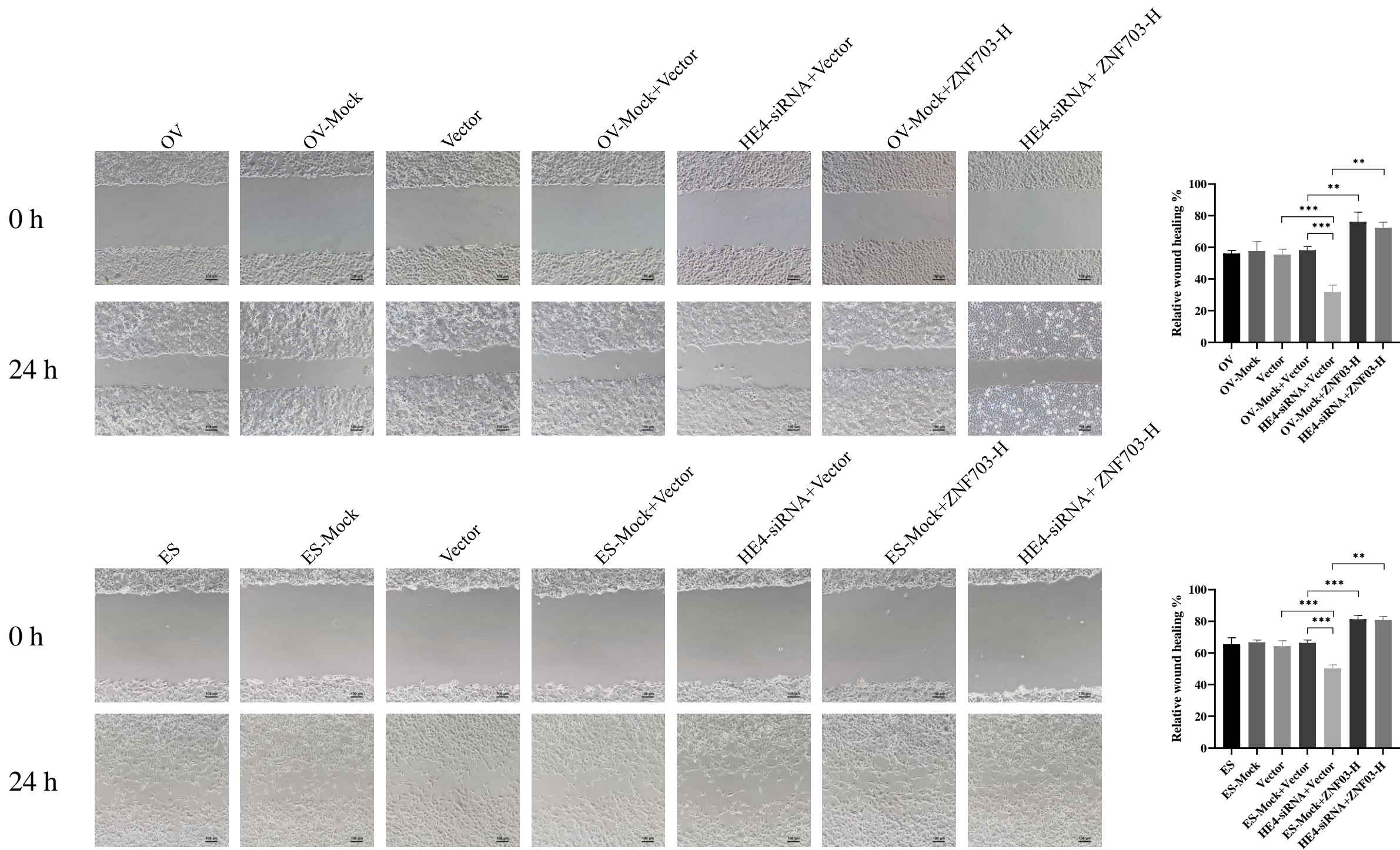

**c**

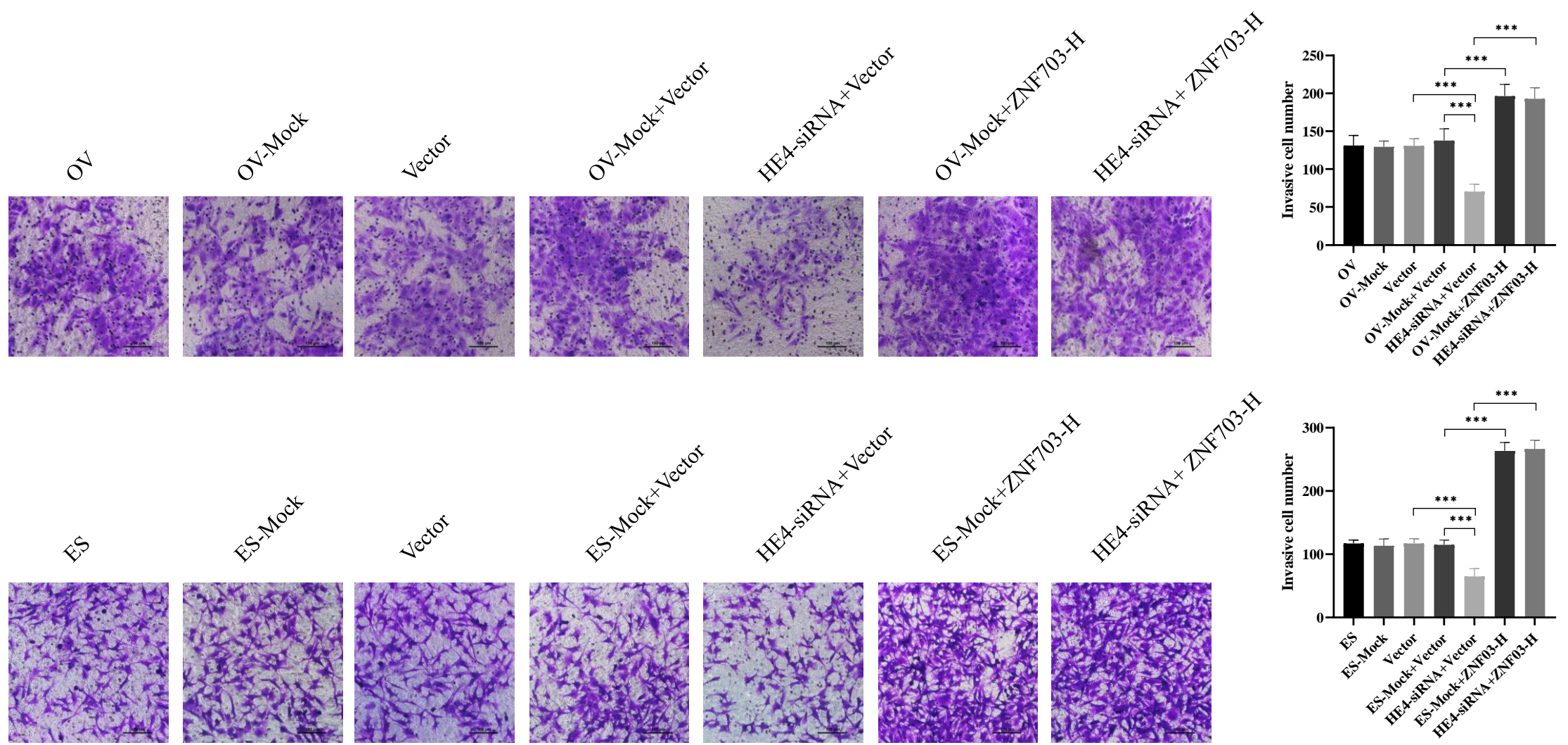

Supplement: Supplementary file 3 — Additional file 3: Figure S3. HE4 impacts on the functional activity of ZNF703. a MTT assays showed HE4 knockdown inhibited cell growth of OVCAR3 and ES-2 cells, while ZNF703 overexpression could restore promoted cell growth. b. HE4 knockdown inhibited cell migration in OVCAR3 and ES-2 cell lines, which could be restored by overexpressed ZNF703 (× 100). c. HE4 knockdown inhibited cell invasion in OVCAR3 and ES-2 cell lines, which could be restored by overexpressed ZNF703 (× 200). Data are presented as mean ± SD. *, P < 0.05; **, P < 0.01; ***, P < 0.001. [file 13046_2020_1770_MOESM3_ESM.pdf]

**a**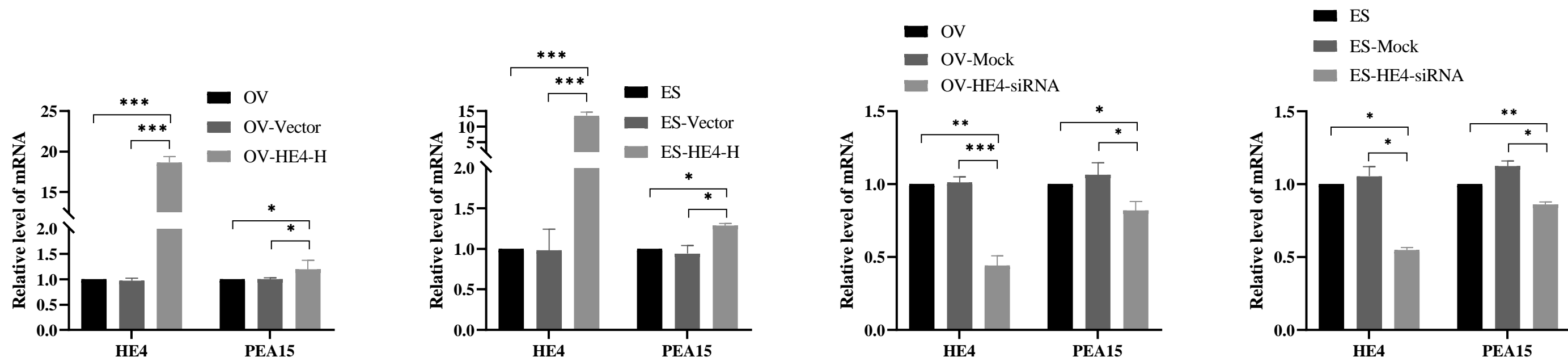**b**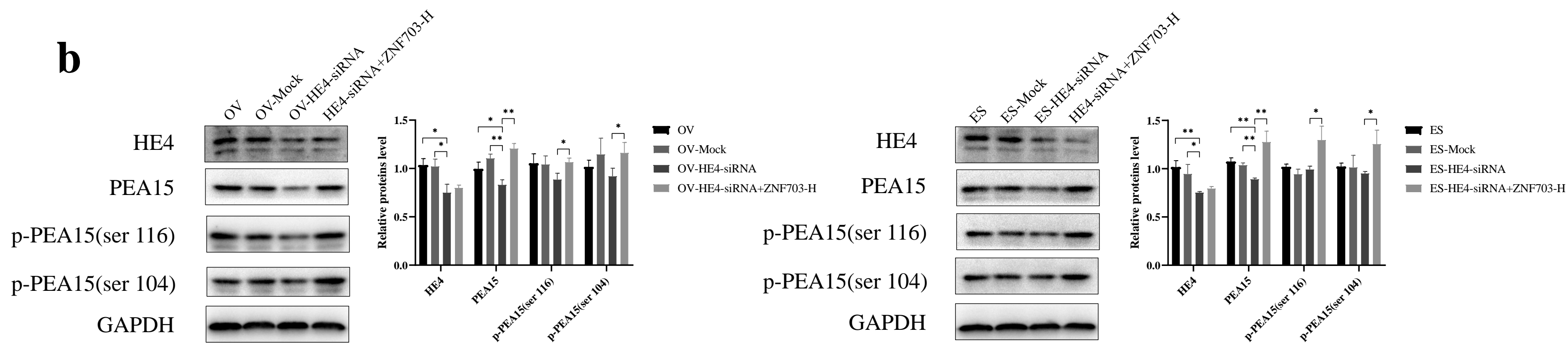**c**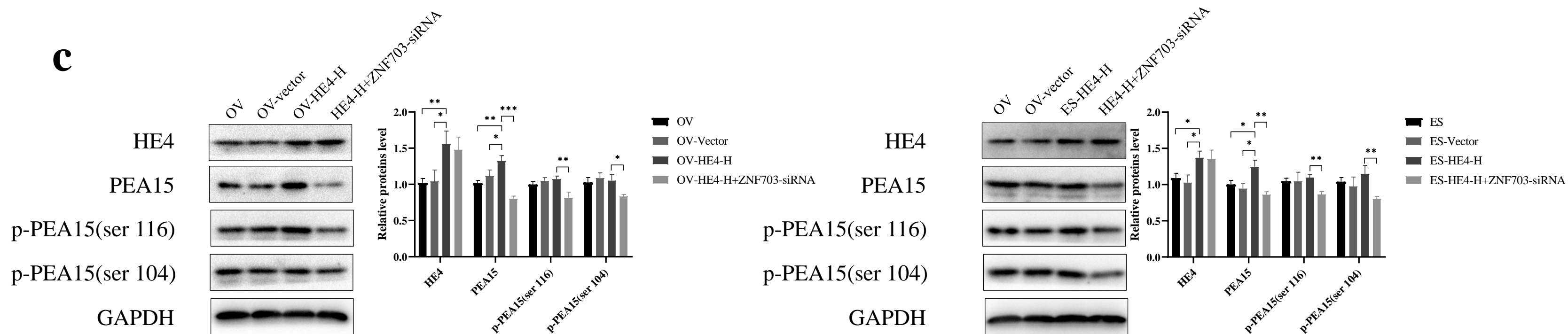

Supplement: Supplementary file 4 — Additional file 4: Figure S4.The effects of HE4 on the ZNF703-dependent regulation of PEA15. a The PEA15 mRNA levels after HE4 siRNA knockdown or overexpression in OVCAR3 and ES-2 cells by qRT-PCR (2-ΔΔCт). b The PEA15 protein levels after HE4 siRNA knockdown with or without ZNF703 overexpression in OVCAR3 and ES-2 cells by western blot. c The PEA15 protein levels after HE4 overexpression with or without ZNF703 siRNA knockdown in OVCAR3 and ES-2 cells by western blot. Data are presented as mean ± SD. *, P < 0.05; **, P < 0.01; ***, P < 0.001. [file 13046_2020_1770_MOESM4_ESM.pdf]

**a**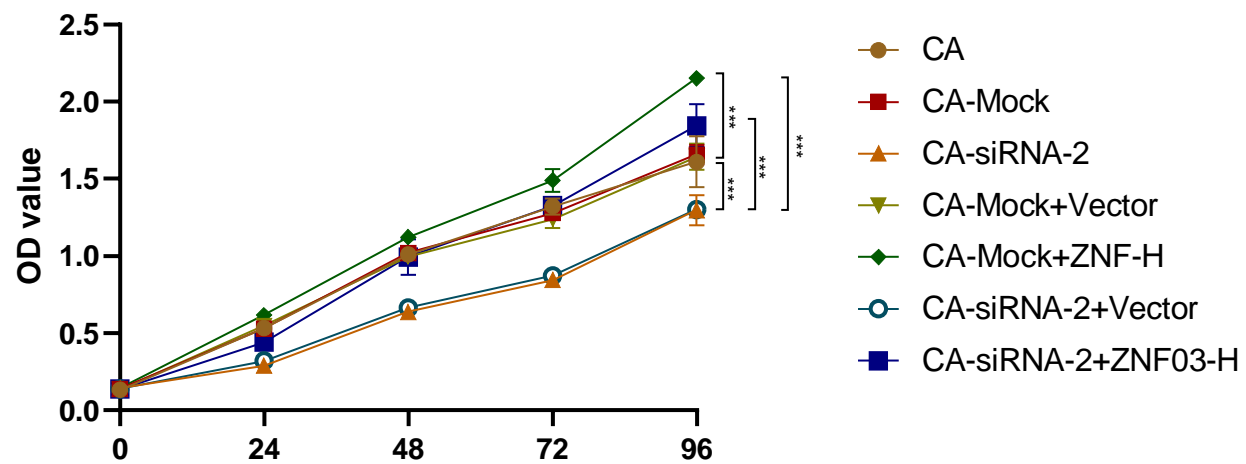**b**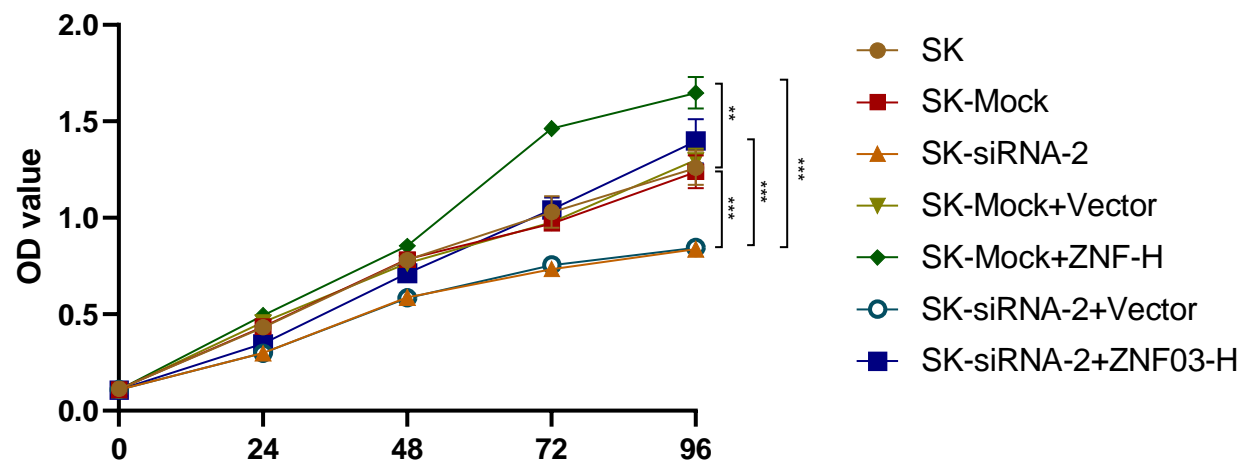

Supplement: Supplementary file 6 — Additional file 6: Figure S6. The effects of overexpression ZNF703 after ZNF703 siRNA knockdown on cell proliferation in cell lines. a MTT assays showed that the overexpression of ZNF703 could rescue the inhibitory effects of ZNF703 knockdown on cell proliferation in CAOV3 cells. b MTT assays showed that the overexpression of ZNF703 could rescue the inhibitory effects of ZNF703 knockdown on cell proliferation in SKOV3 cells. Data are presented as mean ± SD. *, P < 0.05; **, P < 0.01; ***, P < 0.001. [file 13046_2020_1770_MOESM6_ESM.pdf]

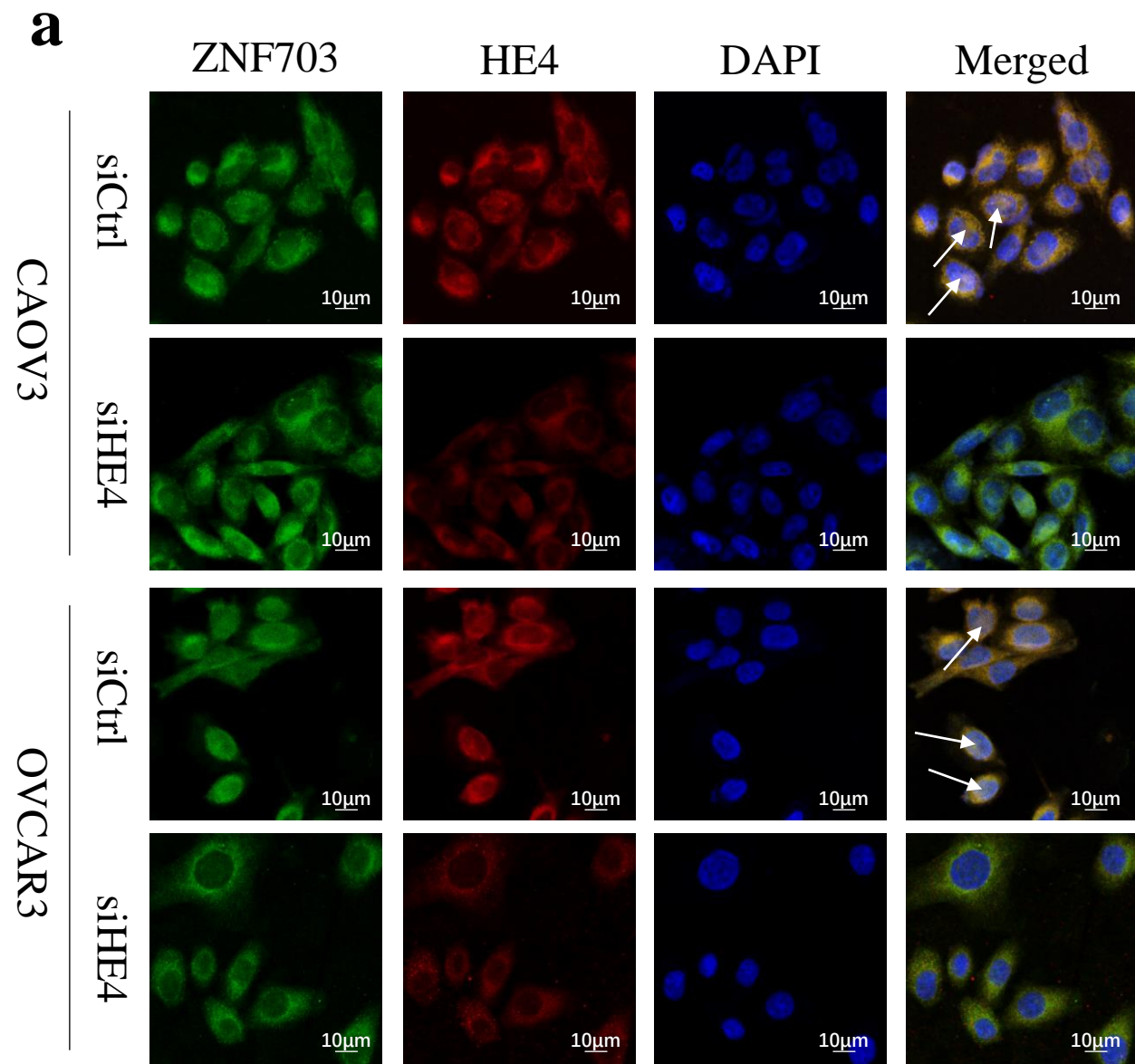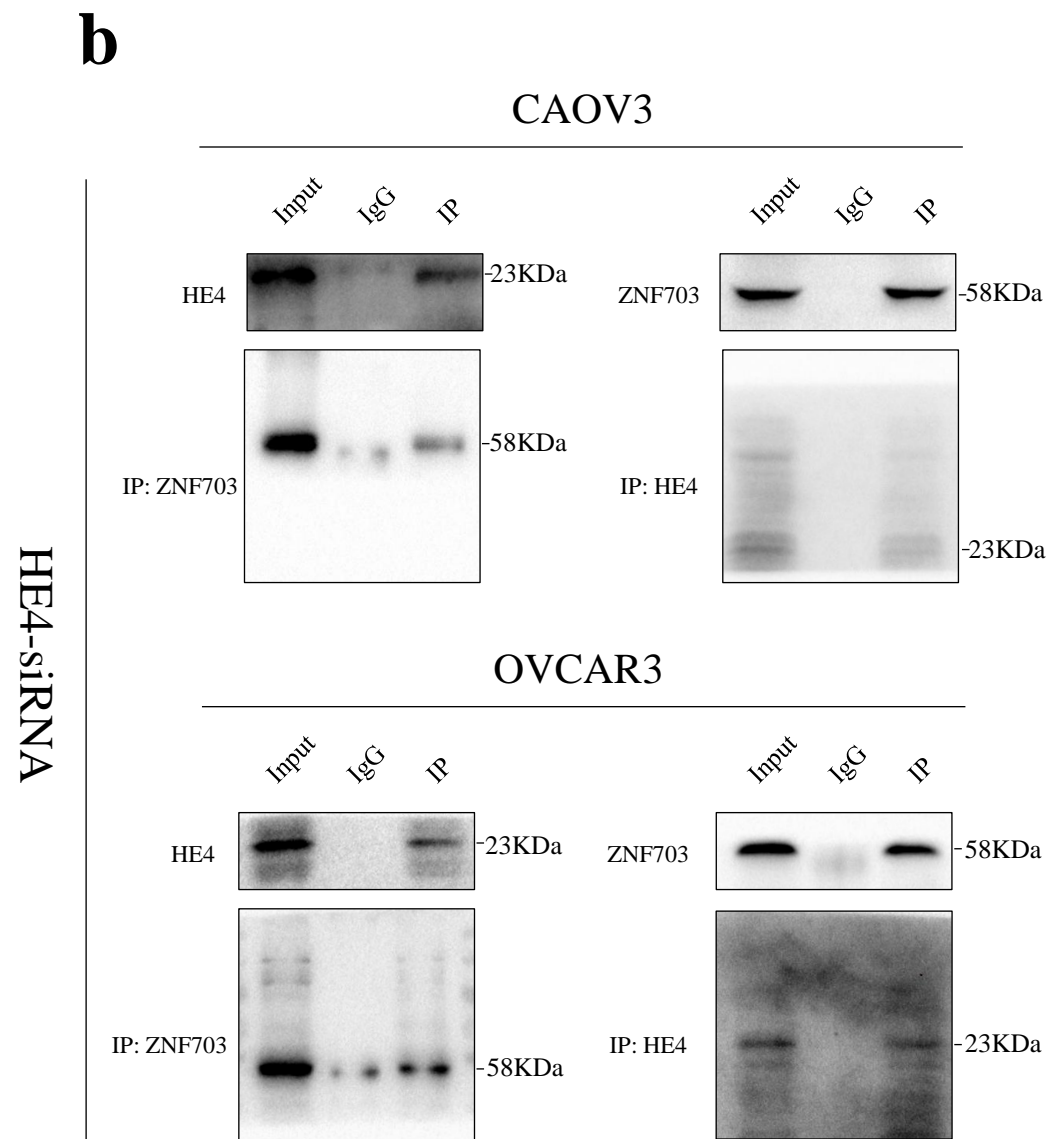

Supplement: Supplementary file 7 — Additional file 7: Figure S7. The interaction of ZNF703 and HE4 during HE4 siRNA knockdown. a The immunofluorescence co-localization of ZNF703 and HE4 was performed in CAOV3 and OVCAR3 cells transfected with HE4 siRNA or control (The green color was for ZNF703 and the red color was for HE4, DAPI was blue, the colocalization was yellow)(× 800). The arrow indicated the co-localization in the nucleus. b The co-immunoprecipitation analysis of ZNF703 and HE4 was performed in ovarian cancer cells transfected with HE4 siRNA (The upper two were performed in CAOV3 cell line and the lower two were performed in OVCAR3 cell line. The corresponding molecular weight was added to the right side). [file 13046_2020_1770_MOESM7_ESM.pdf]

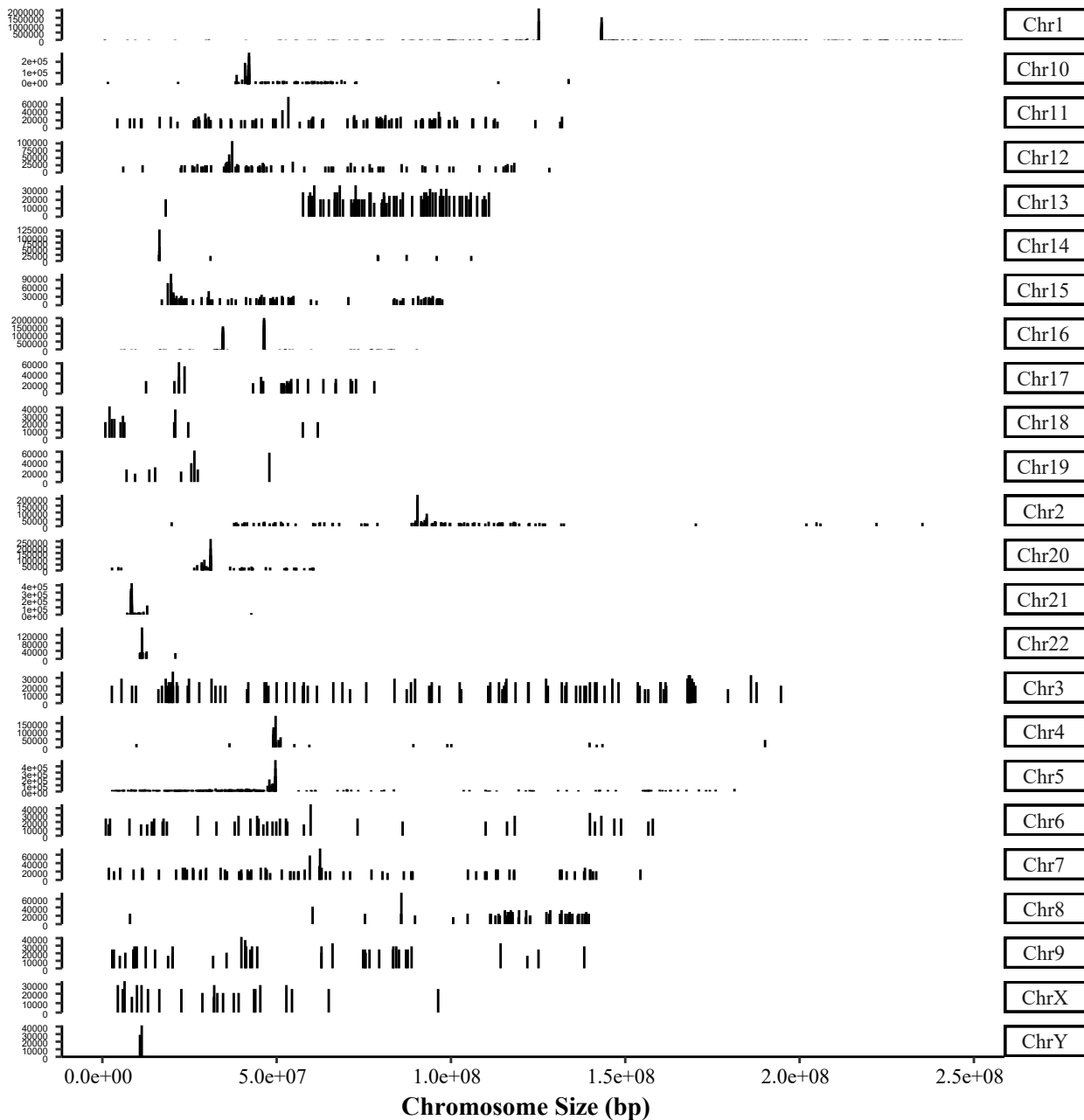

Supplement: Supplementary file 10 — Additional file 10. : Original Figs. 5a-e with each individual. [file 13046_2020_1770_MOESM10_ESM.zip › Additional file 10 Original Figures 5a.pdf]

OH\_IP

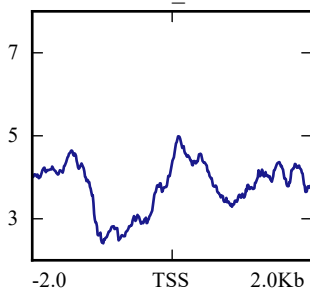

OH\_Input

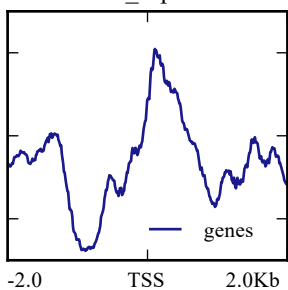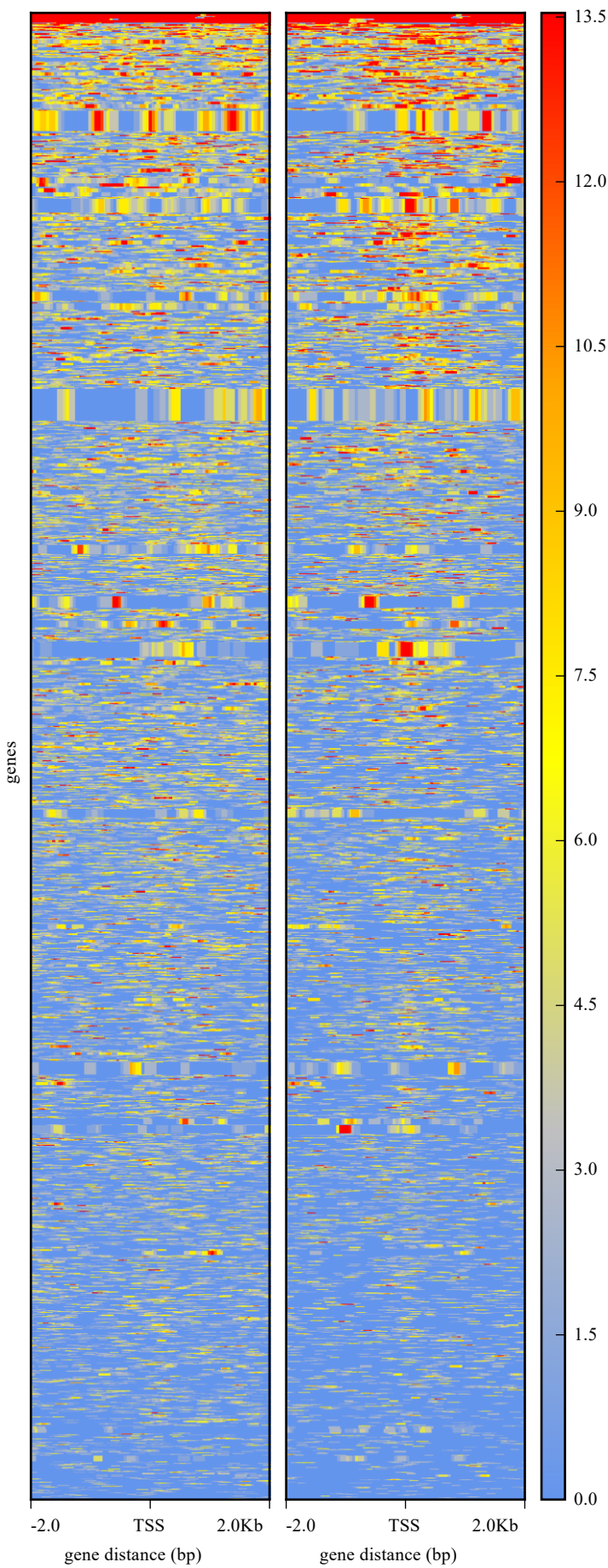

Supplement: Supplementary file 10 — Additional file 10. : Original Figs. 5a-e with each individual. [file 13046_2020_1770_MOESM10_ESM.zip › Additional file 10 Original Figures 5b.pdf]

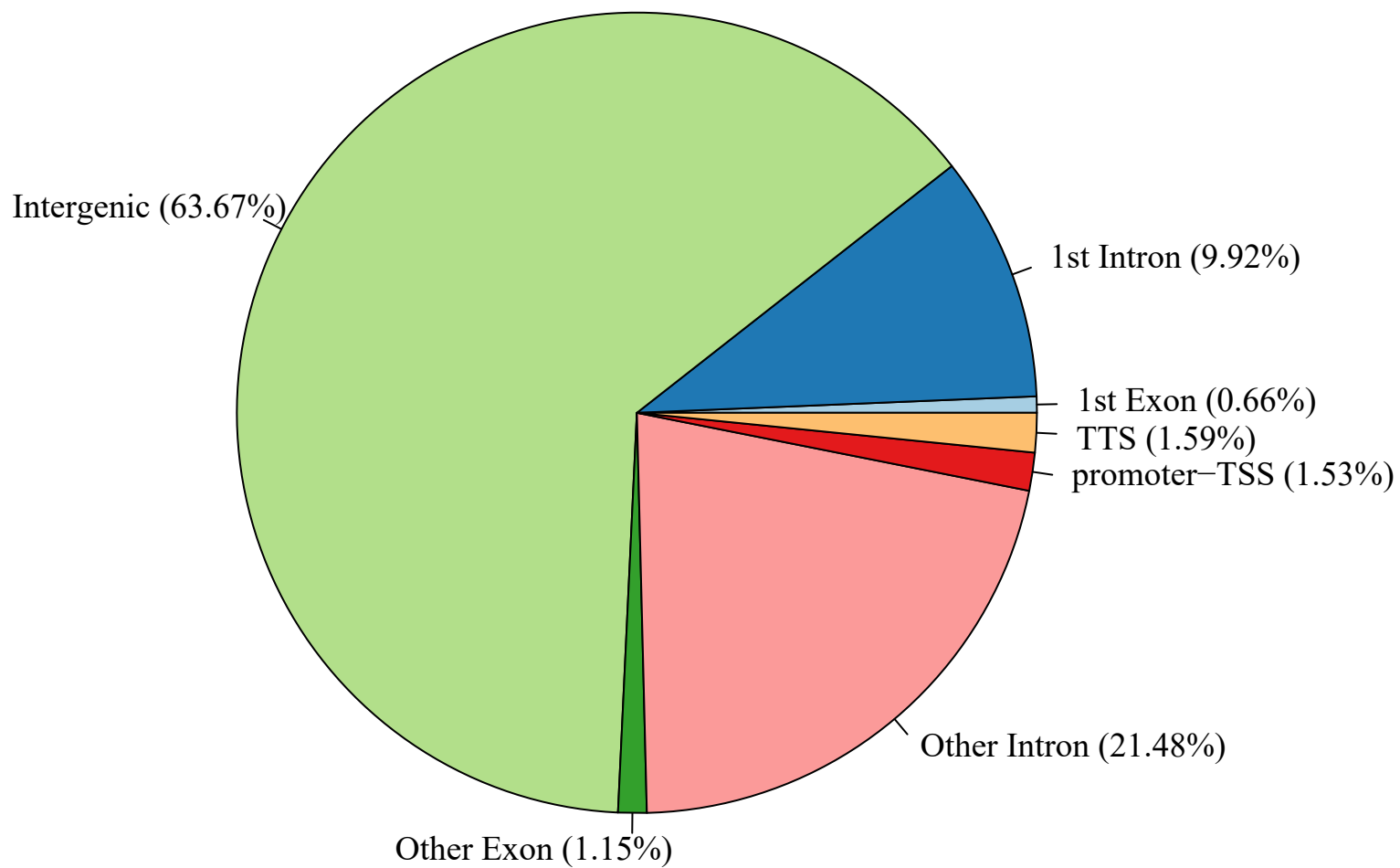

Supplement: Supplementary file 10 — Additional file 10. : Original Figs. 5a-e with each individual. [file 13046_2020_1770_MOESM10_ESM.zip › Additional file 10 Original Figures 5c.pdf]

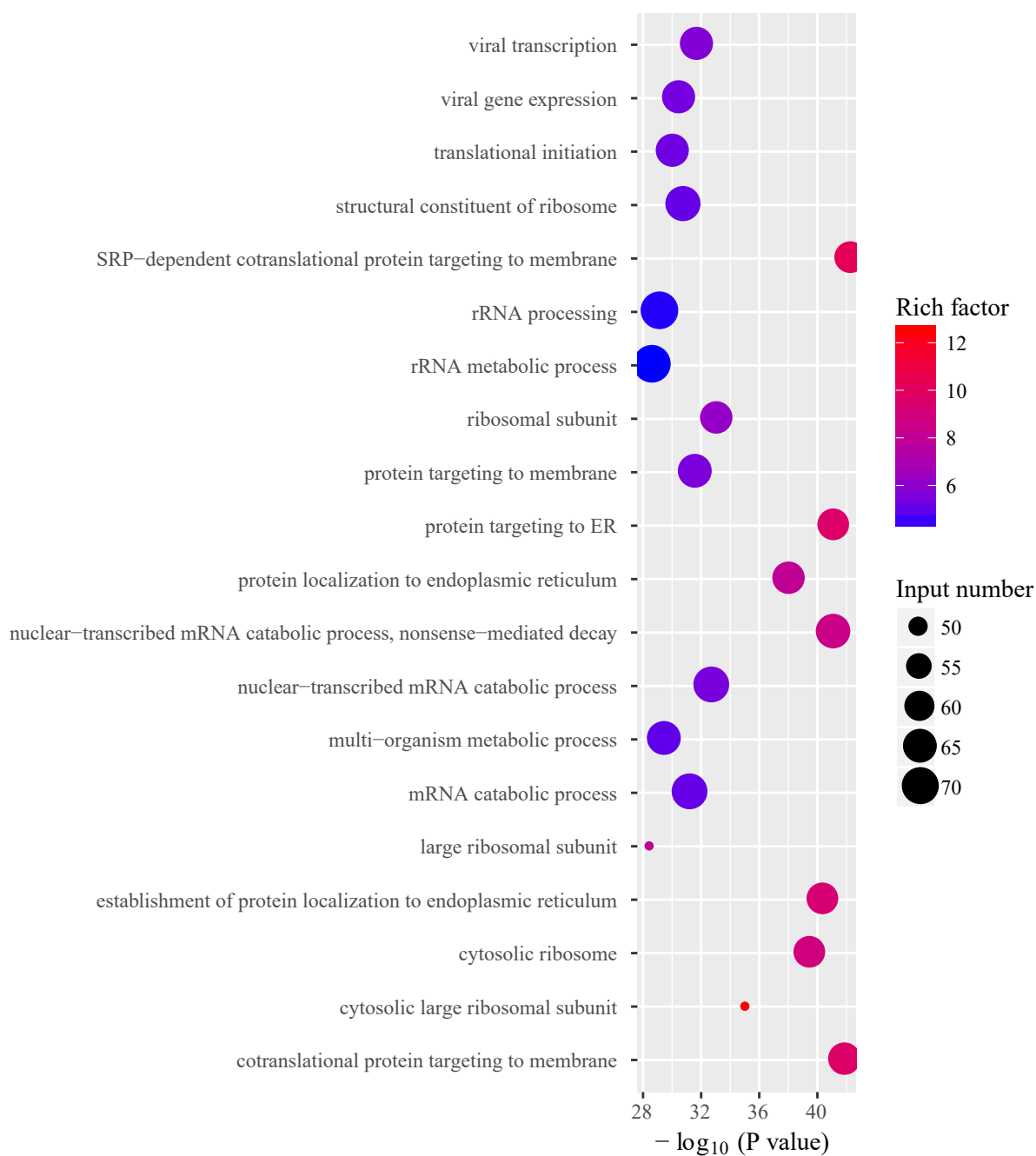

Supplement: Supplementary file 10 — Additional file 10. : Original Figs. 5a-e with each individual. [file 13046_2020_1770_MOESM10_ESM.zip › Additional file 10 Original Figures 5d.pdf]

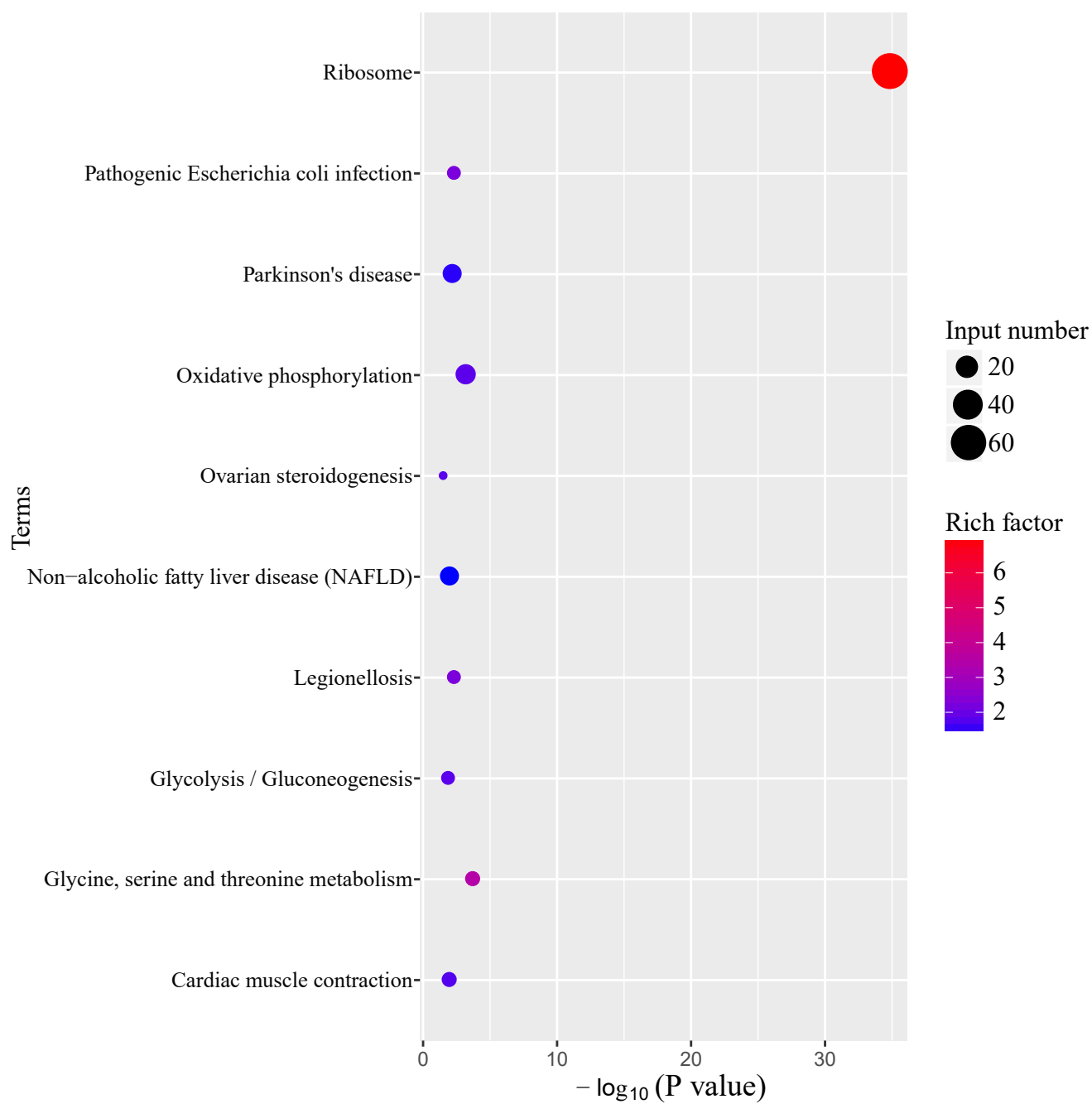

Supplement: Supplementary file 10 — Additional file 10. : Original Figs. 5a-e with each individual. [file 13046_2020_1770_MOESM10_ESM.zip › Additional file 10 Original Figures 5e.pdf]
