# Supplementary material for: ZNF703 promotes tumor progression in ovarian cancer by interacting with HE4 and epigenetically regulating PEA15
Source: J Exp Clin Cancer Res. 2020 Nov 27;39:264. doi: 10.1186/s13046-020-01770-0 (PMC7693506; doi:10.1186/s13046-020-01770-0)
Supplement: Supplementary file 5 — Additional file 5: Figure S5.The expression of PEA15 in clinical specimens and the correlation with ZNF703 or HE4. a PEA15 expression in ovarian tissues samples (Upper left: ovarian malignant tumor, upper right: ovarian borderline tumor, lower left: ovarian benign tumor, lower right: ovarian normal tissue) (× 400, upper right × 200). b Immunohistochemistry staining scores of PEA15 in ovarian tissues samples. c Overall survival analysis according to PEA15 expression in IHC (P = 0.032). d The correlation between ZNF703 and PEA15 expression with Scatter plot in in clinical specimens. e The correlation between PEA15 and HE4 expression with Scatter plot in in clinical specimens. Data are presented as mean ± SD. *, P < 0.05; **, P < 0.01; ***, P < 0.001. Table S2 Expression of PEA15 in different types of ovarian tissue. Table S3 The correlation between ZNF703 and PEA15 expression in ovarian cancer. Table S4 The correlation between PEA15 and HE4 expression in ovarian cancer. [file 13046_2020_1770_MOESM5_ESM.pdf]

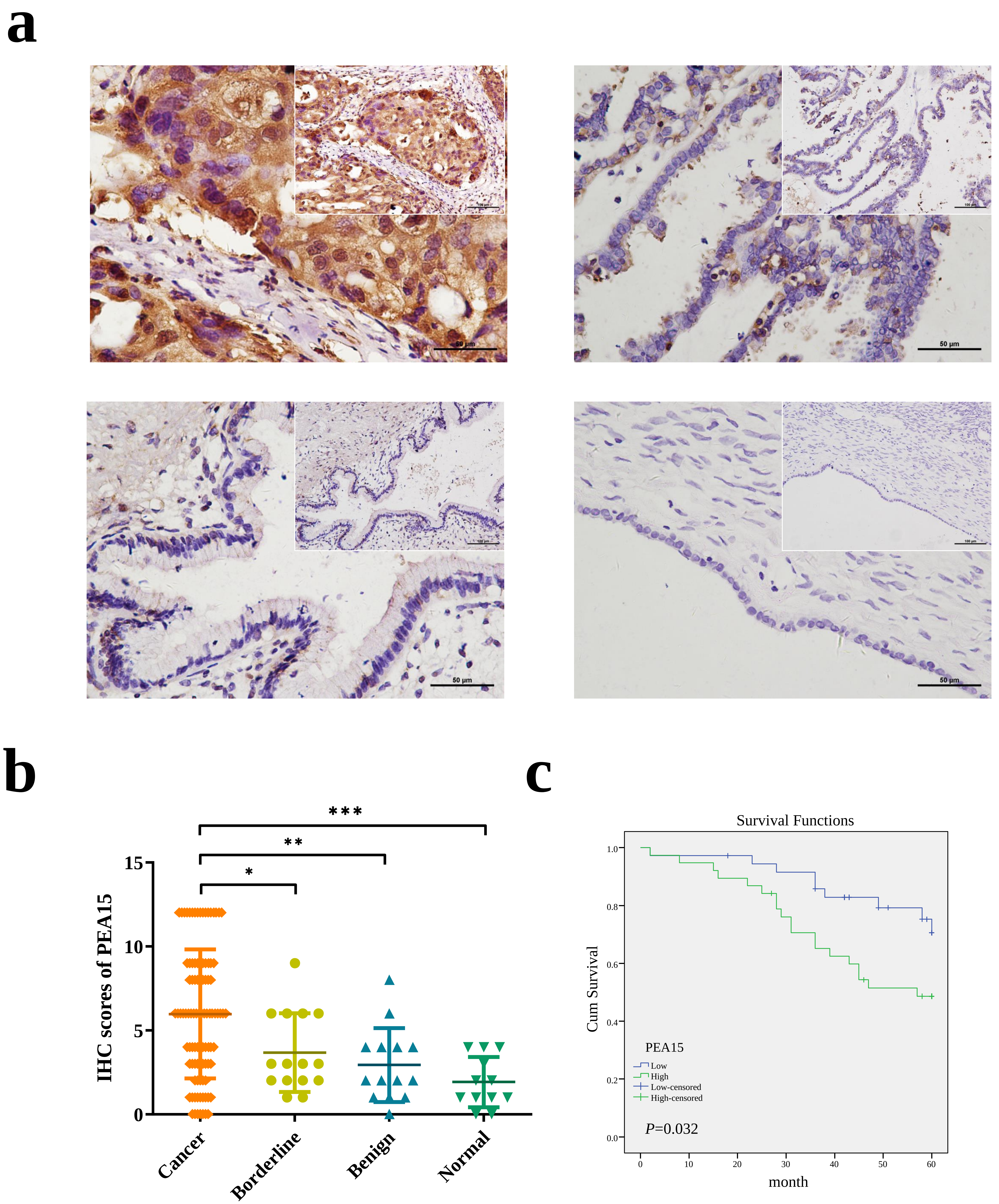
a
50 μm
50 μm
50 μm
50 μm
b
***
**
*
IHC scores of PEA15
15
10
5
0
Cancer
Borderline
Benign
Normal
c
Survival Functions
Cum Survival
1.0
0.8
0.6
0.4
0.2
0.0
0
10
20
30
40
50
60
month
PEA15
Low
High
Low-censored
High-censored
P=0.032

Table S2 Expression of PEA15 in different types of ovarian tissue

Group	Cases	Low		High		Positive rate(%)	High Positive rate(%)
		-	+	++	+++		
Malignant	98	21	20	29	28	78.6[a,b]	58.2[c,d]
Borderline	15	6	4	4	1	60[e,f]	33.3[g,h]
Benign	14	8	4	2	0	42.9	14.3
Normal	12	8	4	0	0	33.3	0

Note: a, malignant vs. benign (**, P=0.008); b, malignant vs. normal (***, P=0.002); c, malignant vs. benign (***, P=0.002); d, malignant vs. normal (***, $P < 0.001$); e, borderline vs. benign ($P = 0.356$); f, borderline vs. normal ($P = 0.168$); g, borderline vs. benign ($P = 0.390$); h, borderline vs. normal (*, $P = 0.047$).

Table S3 The correlation between ZNF703 and PEA15 expression in ovarian cancer (n=98)

ZNF703	PEA15				case
	-	+	++	+++	
-	3	6	3	3	15
+	5	9	6	4	24
++	7	4	9	8	28
+++	6	1	11	13	31
**case**	21	20	29	28	98

d

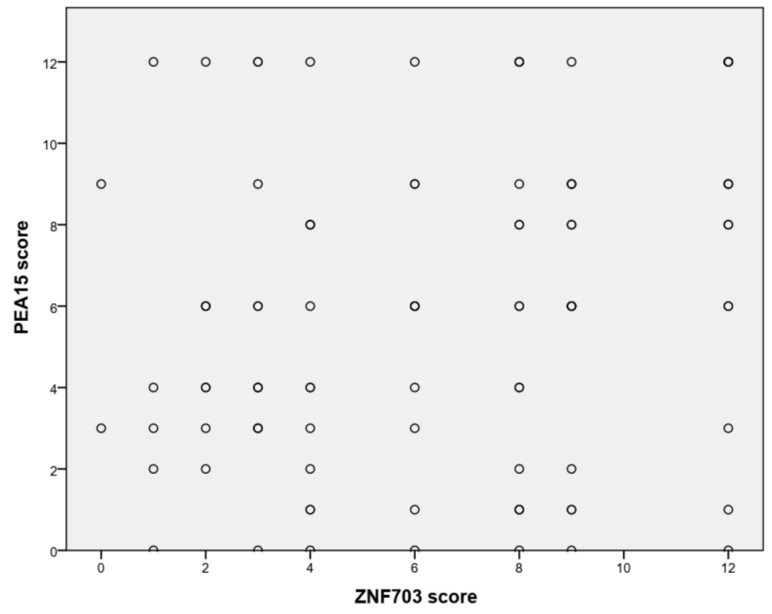

Spearman correlation coefficient Rs=0.237, *P*=0.019

Table S4 The correlation between HE4 and PEA15 expression in ovarian cancer (n=98)

HE4	PEA15				case
	-	+	++	+++	
-	5	3	3	3	14
+	2	8	6	2	18
++	4	3	9	2	18
+++	10	6	11	21	48
**case**	21	20	29	28	98

e

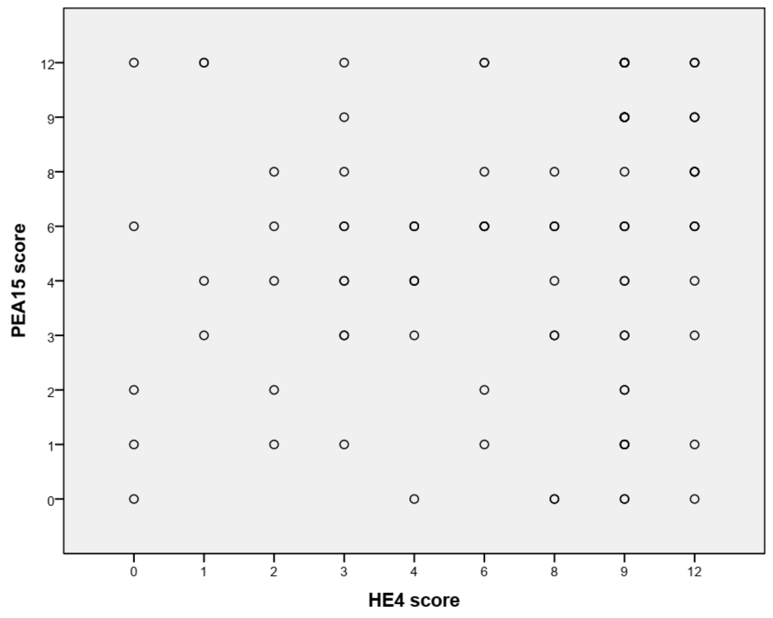

Spearman correlation coefficient Rs=0.203, *P*=0.045
