# Supplementary material for: ZNF703 promotes tumor progression in ovarian cancer by interacting with HE4 and epigenetically regulating PEA15
Source: J Exp Clin Cancer Res. 2020 Nov 27;39:264. doi: 10.1186/s13046-020-01770-0 (PMC7693506; doi:10.1186/s13046-020-01770-0)
Supplement: Supplementary file 8 — Additional file 8: Table S5. ZNF703 binding DNA sites (Displaying some of results including PEA15). Table S6. Primers for qRT-PCR (5′ to 3′). Table S7. siRNA sequences (GenePharma, Shanghai, China). Table S8. Primers for ChIP-PCR (5′ to 3′). Table S9. Antibodies used in the manuscript. Table S10. Reagents and Kits used in this work. [file 13046_2020_1770_MOESM8_ESM.docx]

**Additional file 8: Other Supplemental Tables**

**Table S5.** ZNF703 binding DNA sites (Displaying some of results including PEA15)

| PeakID | Chr & strand | Start | End | Annotation | Distance to TSS | Nearest Gene Name |
| --- | --- | --- | --- | --- | --- | --- |
| Peak_1054 | chr3+ | 87312250 | 87312538 | Intergenic | -10874 | KRT8P25 |
| Peak_1061 | chr3+ | 102506237 | 102506525 | Intergenic | 71366 | ZPLD1 |
| Peak_1167 | chr5+ | 6263643 | 6263963 | Intergenic | -26054 | HMGB3P3 |
| Peak_1199 | chr5+ | 15874843 | 15875131 | Intergenic | -60195 | MIR887 |
| Peak_1609 | chr6+ | 109948472 | 109948760 | Intergenic | -30497 | GPR6 |
| Peak_1681 | chr7+ | 118121229 | 118121702 | Intergenic | -62567 | LSM8 |
| Peak_1701 | chr8- | 111248721 | 111249009 | Intergenic | -12662 | LINC01609 |
| Peak_178 | chr1+ | 182828576 | 182828907 | Intergenic | -10628 | DHX9 |
| Peak_323 | chr10- | 57407293 | 57407581 | Intergenic | -102878 | MIR3924 |
| Peak_1693 | chr8+ | 7931983 | 7932271 | intron | 5790 | ZNF705B |
| Peak_1646 | chr7- | 42215340 | 42215628 | intron | 21529 | GLI3 |
| Peak_136 | chr1- | 152362478 | 152362766 | intron | -2616 | FLG2 |
| Peak_150 | **chr1+** | **160199305** | **160199593** | **intron** | **-5888** | **PEA15** |
| Peak_1012 | chr22+ | 20936437 | 20936725 | intron | 19155 | CRKL |
| Peak_1104 | chr3+ | 168531882 | 168532204 | intron | 12436 | EGFEM1P |
| Peak_1174 | Chr5- | 9147327 | 9147615 | intron | -93576 | MIR4636 |
| Peak_1115 | chr3- | 194660734 | 194661022 | intron | 11599 | LSG1 |
| Peak_137 | chr1- | 152566681 | 152566969 | promoter-TSS | -53 | LCE3E |
| Peak_1643 | chr7- | 39855386 | 39855712 | promoter-TSS | -493 | RWDD4P2 |
| Peak_502 | chr12- | 80561482 | 80561770 | promoter-TSS | -66 | AKIRIN1P1 |
| Peak_49 | chr1+ | 99860766 | 99861054 | promoter-TSS | -300 | AGL |
| Peak_628 | chr15+ | 30517675 | 30518043 | TTS | 1780 | AC026150.6 |
| Peak_588 | chr13- | 109100605 | 109100893 | TTS | 1170 | MYO16-AS2 |

**For peak 150 sequences:** TAAAGAAAACCAATGTTGTGAGGCACAATAGTCCTGCACATGGGTATACACACTCAATCATAGAATCATAGCACAGAGATTCCCTGTTTCAGGAGATGATTTTGTTCTCAGCTACATTCCCAACACCTAGCACTCGCTGTTGAATGAGTAACTGAAAGAACAAAGAGAAACGTGAGAAACATTGGCACTTACCAGGTATACTTGCCTTATTTTAGAAATGTAAGATCTGAGGGCCAGAGAGAAGTGACTTGACCTTGAACACAAGGATCTGATCCAGGATCAGAACAGG (The highlights was the sequence which potential binding site verified by ChIP-PCR with PEA15-2 primers)

**Table S6**. Primers for qRT-PCR (5’ to 3’)

| Gene name | sequence |
| --- | --- |
| *ZNF703* | F: TGGATCCTAGCAAGTCCGG  R: TGGTAACCTCCCAAGCAATAG |
| *PEA15* | F: CCCGCCGTCCTGACCTACTC  R: TTGGTGTCCAGCTCATCCTCCTC |
| *AGL* | F: GAATCCCAGCCACACCAAGAGATG  R: TTCCAGCAACCAGCGAACAGC |
| *HE4* | F: CCTGCTGCTGTTCGGCTTCAC  R: CTCTTGCGTGCAGTTCTGGTCAG |
| *GAPDH* | F: ACAACTTTGGTATCGTGGAAGG  R: GCCATCACGCCACAGTTTC |
| *Actin* | F: CAGCAAGCAGGAGTATGACG  R: TTAGGATGGCAAGGGACTTC |

**Table S7.** siRNA sequences (GenePharma, Shanghai, China)

| Name | sequence |
| --- | --- |
| siRNA control | UUCUCCGAACGUGUCACGUTT  ACGUGACACGUUCGGAGAATT |
| si-ZNF703-1 | GGAUCUAACCCAAGGACACTT  GUGUCCUUGGGUUAGAUCCTT |
| si-ZNF703-2 | GGACAAGAAAGACCAGGAGTT  CUCCUGGUCUUUCUUGUCCTT |
| si-ZNF703-3 | AGAAACUUCUGGCCGCCUATT  UAGGCGGCCAGAAGUUUCUTT |
| si-HE4 | AGGUGAACAUUAACUUUCCTT  GGAAAGUUAAUGUUCACCUTT |

**Table S8**. Primers for ChIP-PCR (5’ to 3’)

| Name | sequence |
| --- | --- |
| AGL-1-Qchip-F | TCCCTTCCCACTTCCACG |
| AGL-1-Qchip-R | AGGCAACTGAACCCGACT |
| AGL-2-Qchip-F | GCCTTCAGAACTCCTGTT |
| AGL-2-Qchip-R | ATGGGACTATTATCTCACCA |
| AGL-3-Qchip-F | ACTCCAAGTCAGAGGTTCCC |
| AGL-3-Qchip-R | CTCACCATACTGAGAAAGCTAGT |
| PEA15-1-Qchip-F | TCCCTGTTTCAGGAGATG |
| PEA15-1-Qchip-R | CAGCGAGTGCTAGGTGTT |
| PEA15-2-Qchip-F | **TGTGAGGCACAATAGTCCTG** |
| PEA15-2-Qchip-R | **TAGGTGTTGGGAATGTAGC** |
| PEA15-3-Qchip-F | TCCCAACACCTAGCACTC |
| PEA15-3-Qchip-R | CCTGGATCAGATCCTTGTG |

**Table S9. Antibodies** **used in the manuscript**

| Antibody | Catalogue NO. | Company |
| --- | --- | --- |
| PI3K | 4292S | Cell Signaling Technology (Beverly, MA) |
| p-PI3K | 4228S | Cell Signaling Technology (Beverly, MA) |
| AKT | 4691S | Cell Signaling Technology (Beverly, MA) |
| p-AKT | 4060S | Cell Signaling Technology (Beverly, MA) |
| Cyclin D1 | 2978S | Cell Signaling Technology (Beverly, MA) |
| PCNA | 2586S | Cell Signaling Technology (Beverly, MA) |
| Bax | 5023S | Cell Signaling Technology (Beverly, MA) |
| PEA15 | 2780S | Cell Signaling Technology (Beverly, MA) |
| IgG | 5145S | Cell Signaling Technology (Beverly, MA) |
| ZNF703 | sc-271896 | Santa Cruz Biotechnology (Santa Cruz, CA) |
| ZNF703X | sc-271896 X | Santa Cruz Biotechnology (Santa Cruz, CA) |
| HE4 | 200828 | Abcam (Cambridge, UK) |
| HE4 | DF8160 | Affinity Biosciences (Cincinnati, OH, USA） |
| Bcl-2 | 12789-1-AP | Proteintech(Wuhan, China) |
| MMP2 | 10373-2-AP | Proteintech(Wuhan, China) |
| MMP9 | 10375-2-AP | Proteintech(Wuhan, China) |
| Lamin B1 | 12987-1-AP | Proteintech(Wuhan, China) |
| GAPDH | TA-08 | ZSGB-BIO(Beijing, China) |
| p-PEA15(Ser104) | YP0701 | ImmunoWay Biotechnology (Plano, TX, USA) |
| p-PEA15(Ser116) | YP0669 | ImmunoWay Biotechnology (Plano, TX, USA) |
| Goat anti-mouse IgG (H+L), FITC conjugate | SA00003-1 | Proteintech(Wuhan, China) |
| Goat anti-mouse IgG (H+L), TRITC conjugate | SA00007-1 | Proteintech(Wuhan, China) |
| Goat anti-rabbit IgG (H+L), FITC conjugate | SA00003-2 | Proteintech(Wuhan, China) |
| Goat anti-rabbit IgG (H+L), TRITC conjugate | SA00007-2 | Proteintech(Wuhan, China) |

**Table S10.** Reagents and Kits used in this work

| Name | Catalogue NO. | Company |
| --- | --- | --- |
| UltraSensitive SP | KIT-9720 | MaXim (Fujian, China) |
| Triton X-100 | P0096 | Beyotime (Shanghai，China） |
| 1640 | 01-100-1A | Biological Industries (Beit-Haemek, Israel). |
| McCoy’s 5A | 01-075-1A | Biological Industries (Beit-Haemek, Israel). |
| Fetal bovine serum | 04-001-1A | Biological Industries (Beit-Haemek, Israel). |
| Lipo3000 | L3000015 | Thermo Fisher (MA, USA). |
| RNAiso Plus | 9109 | Takara (Tokyo, Japan) |
| PrimeScript RTreagent Kit | TAKARA047A | Takara (Tokyo, Japan) |
| Premix Ex Taq II Kit | TAKARA820A | Takara (Tokyo, Japan) |
| RIPA | P0013B | Santa Cruz Biotechnology (Santa Cruz, CA) |
| Chemiluminescent HRP Substrate | P90720 | Thermo Fisher (MA, USA). |
| MTT | M8180 | Solarbio (Beijing, China) |
| DMSO | D8370 | Solarbio (Beijing, China) |
| [Annexin-V-APC/7AAD](http://www.baidu.com/link?url=03e7Z36w9Xq_7WlG8tNdw1Y3wGQnAzJ5p9EqA2EE8ccMO-0l6MhoVXgIm-0cLSBjffA9ZnCOoS8sGiCowQKm3q) | 550474 | BD Biosciences(New York, USA) |
| Annexin-V-FITC/PI | KGA107 | KeyGen Biotech (Nanjing, China) |
| Cell cycle Detection Kit | KGA512 | KeyGen Biotech (Nanjing, China) |
| Matrigel | 356234 | BD Biosciences(New York, USA) |
| Protein A/G PLUS-Agarose beads | sc-2003 | Santa Cruz Biotechnology (Santa Cruz, CA) |
| DAPI | 4083S | Cell Signaling Technology (Beverly, MA) |
| Nuclear and Cytoplasmic Protein Extraction Kit | P0027 | Beyotime (Shanghai，China） |
| SimpleChIP® Plus Enzymatic Chromatin IP Kit | 9004S | Cell Signaling Technology (Beverly, MA) |
| Dual Luciferase Reporter Assay System | E1910 | Promega (Madison, WI, USA) |
