# Supplementary material for: ZNF703 promotes tumor progression in ovarian cancer by interacting with HE4 and epigenetically regulating PEA15
Source: J Exp Clin Cancer Res. 2020 Nov 27;39:264. doi: 10.1186/s13046-020-01770-0 (PMC7693506; doi:10.1186/s13046-020-01770-0)
Supplement: Supplementary file 9 — Additional file 9. : The details regarding the plasmids employed in the luciferase experiments. [file 13046_2020_1770_MOESM9_ESM.docx]

**The details regarding the plasmids employed in the luciferase experiments of Fig. 6h.**

The plasmids employed in the luciferase experiments were synthesized by GeneChem (Shanghai, China). The PEA15 promoter fragments (2kb upstream of TSS) were digested with restriction endonucleases KpnI and XhoI (Thermo Fisher Scientific).

It recognizes the sequences GGTACC^CTCGAG. The enhancer-WT or enhancer-Mut sequences were digested with restriction endonucleases XbaI and XbaI. They recognize the sequences TCTAGA^TCTAGA. Then, they were ligated into the pGL3-basic plasmid (Promega).After that, the construction of the luciferase reporter systems were completed, named: PEA15 promoter-Luciferase, PEA15 promoter-Luciferase-enhancer-WT and PEA15 promoter-Luciferase-enhancer-Mut.

**The PEA15 promoter sequence was**: AAACAAAACGAAACAAAACAAACTAGCAAAATAGGCTGTCCCCAGTGCAAGTGCAGGTGCCAGAACATTTCTCTATCGATAGGTACCTCTGTTGCCAGGTTGGAGTGCAGTGGTGAGATCTCGGTTCACTGCAACCTCCGACTCCCGGGTTCAAGAGATTCTCCTATCTCAGCCTCCCAGGTAACTGGGACTACAGGTGCCCACCACCACACTCAGCTAACTTTTTTGTAGTTTTAGTAGAGATGGGGTTTCACCATGTTGGTCAGGCTAGTCTTGAACTCCTGACCTCAGGTGATCCACCTGCCTCAGCCTCCCAAAGTGCTGGGATTACAGGCGTGAGCCACCACGCCAAGCCAACATTATATTTTTCATGGATCAGGTGTAAAGTTGTTGCTGAGAGACTTAGAGGATTTCTAAGTACGGAAAGATGAGACCTGAGATTAGATATATTTTCACTCTCCACAGACAAGTCCAGGAAGTGTTCCTAGAGAATGTGGCAGGGGTGTAGAAAGCAGAAACTATGTATTCATTCCAACTTGTAGCTATTTAATAAATGTTTAATAAGTAGTATGGGCACTAAGCTAGTCTTACAATAATATTGTGGAGCTTACTGTCTAGCATAGGTCAGTGGAAGGCCCCGGTCTAAATAATAGCTCCTATTTTTTAAGCACCCATCTGTGCCAGTCCCTAGAGACTTACTATTTCTGAATCTCAGAAGAACCCTCAGAAATAGACATTGGCACCCTGATCTGACCGGTGAGGACACTAAGGCTTTCAGGCATAACTTGCCCAAGGAATGGGTGGAACTAGCACGCAGCCCAAGGCCCCTGTGTCTTGTTGCCTCCAGACCTGAGCCTTTCACTTAGAAACTACATGAGTCTCAAAGATGGGTCAACGAAATAGTTTTTGTCCCTACAACACAGAGCAAACATGATATATACAAAGGCAAATGATTAGTGTGTGACAGGGAATGCTGGGGAGAGGAAACAGCAGAGAAATGCTATGAAACCCAGAGCAGCAGGAACTTGGGCCTCTTCTAGGAATGGTGTAGACCCAAGAAAGGAGGGGCTCGTTTGAGGTTTCTGGAGGAGTCTCTGCCAGGTAGGAACTGAGATGAGATGGGTGATCCTGGAAGTGGCACAAACACTGAGGGGAATCCCCCAACCTGGGCCTGCTGGAGTTCCTGGCAGGAAGGACCTACCCAGGGTCAGACTTCTTTCCAGGGCCAAACCCTGCCCAGAGCTGAGGCAAGACCCCTTAGGCTATAGAGATGGGGGTGAAAACAGATGCTTCTCCAATTCTTCCTTTCCTCTTTTCAGTCTGGCTGAGAATCTGGGGGAGGAAAGTCCCTGGGGTAGAGGGGAAGTGAGAGCTGCCACATTGGGCCTGGGCAGAGGGGCAGAGCTAGGAGATGGAGCTAAGTGTGGCTTTGCAAGTTGTCTATATTTGCATGCTGAGCTCACTGCTGCTCTTTTTCTCCCCAGAAAATAAAATTACATCATGGTAGGGGGAAGGCAGGAGTGGGGCATGTTTGGGAAAGGTGAGAGGCACGCAAGAGGGCCTGAAGGCATCATCCTGGAAAGAGAAGATCCAGGGCTCCTCAAGTCCCCATGTGGCCCCAACCCCTCACTCCCAATGTGGATAAGTCTGATCAGTCATTTTCCACTGCGCCCCTGACCCTTAAGGCAAGGCAAGAAGTTTTTACAAATATTAAAGCCAGCAGCTAAGAGAATCCAGCCCCCAAAGGCTTTCCAACCCCCTAAACCAAACCTACTCTGTGCCTCCCAGGGGTCCTAGGCACCGTGGTCATCCAAAGGTCAAAAGGGGAGGAGGGTGCCCTACTGGGTAAGAATTAAATATGAGAATTGTTTCGCAGTTCAGGGGGAAGAGACCGTCTCCGCTCCAGCCCTCCGCCCAATTCTGTCCCCCATCCTAGGAGTCTTCCAGAACCCCAGCCCCTCGGTCCCCCGCCCCCTCCTGCCTCTCCATCCCTCCCCCTCCTCCCGCCATTCCCCGCCCCCCGCCCCTCCCCGGCTCTGACATCACCGGCCAGCCGGGTGGAGTGAGCGGCGCTGGGCTCGGGCTCCGGCTCCGCGGGCGGAAGAGGCGGCGGCGGCGGCAGAAGCGGCGGCGGCGGCTCGAGATCTGCGATCTAAGTAAGCTTGGCATTCCGGTACTGTTGGTAAAGCCACCATGGAAGACGCCAAAAACATAAAGAAAGGCCCGGC

**The PEA15 promoter-Luciferase-enhancer-WT sequence was:** TCTAGATGGCATGGAGGGCATCCTGAGCAAGGGGAAACAGGACGGACATCACACACATTCCCAGAGCAGAGGCTGGATGGGTAGCTCAGGTGCTAGGGCTGACTCCTAAAGAAAACCAATGTTGTGAGGCACAATAGTCCTGCACATGGGTATACACACTCAATCATAGAATCATAGCACAGAGATTCCCTGTTTCAGGAGATGATTTTGTTCTCAGCTACATTCCCAACACCTAGCACTCGCTGTTGAATGAGTAACTGAAAGAACAAAGAGAAACGTGAGAAACATTGGCACTTACCAGGTATACTTGCCTTATTTTAGAAATGTAAGATCTGAGGGCCAGAGAGAAGTGACTTGACCTTGAACACAAGGATCTGATCCAGGATCAGAACAGGGTTTCCTATCCAGTTTTCCTCAGAAAAGAGGGGCCAGTGCACCCAGCTGACCCTCCCGATTCTCTCCCTTTAGGATTTCTCCTCTTTTGTTGTTTAGTTCTCTAGA

**The PEA15 promoter-Luciferase-enhancer-Mut sequence was:** TCTAGATGGCATGGAGGGCATCCTGAGCAAGGGGAAACAGGACGGACATCACACACATTCCCAGAGCAGAGGCTGGATGGGTAGCTCAGGTGCTAGGGCTGACTCCTAAAGAAAACCAATGTGCACTCGCTGTTGAATGAGTAACTGAAAGAACAAAGAGAAACGTGAGAAACATTGGCACTTACCAGGTATACTTGCCTTATTTTAGAAATGTAAGATCTGAGGGCCAGAGAGAAGTGACTTGACCTTGAACACAAGGATCTGATCCAGGATCAGAACAGGGTTTCCTATCCAGTTTTCCTCAGAAAAGAGGGGCCAGTGCACCCAGCTGACCCTCCCGATTCTCTCCCTTTAGGATTTCTCCTCTTTTGTTGTTTAGTTCTCTAGA

The deleted sequence is TGTGAGGCACAATAGTCCTGCACATGGGTATACACACTCAATCATAGAATCATAGCACAGAGATTCCCTGTTTCAGGAGATGATTTTGTTCTCAGCTACATTCCCAACACCTA. It was the potential binding site verified by ChIP-PCR with PEA15-2 primers.
